# Separating Faces in ARMS Metabarcoding Improves Marine Biodiversity Monitoring: A Comparison Across Protocols, Experimental Designs and Photographic Surveys

**DOI:** 10.1111/1755-0998.70188

**Published:** 2026-09-11

**Authors:** Anne Chenuil, Elyna Bouchereau, Térence Legrand, Virgile Calvert, Cécile Chemin, Sandrine Chenesseau, Dorian Guillemain, José Miguel Gutiérrez Ortega, Anne Haguenauer, Michèle Leduc, Frédéric Legendre, Florent Marschal, Christian Marschal, Fatma Mirleau, Marjorie Selva, Laurent Vanbostal, Frédéric Zuberer, Pascal Mirleau, Laetitia Plaisance, Vincent Rossi, Sandrine Ruitton, Emese Meglécz, Vincent Dubut

**Affiliations:** ^1^ Aix Marseille Univ, Avignon Univ, CNRS, IRD, IMBE Marseille France; ^2^ Aix Marseille Univ, Université de Toulon, CNRS, IRD, MIO Marseille France; ^3^ Ifremer, DYNECO‐Dynamiques Des Écosystèmes Côtiers Plouzané France; ^4^ Aix Marseille Univ, CNRS, IRD, INRAE, OSU PYTHEAS Marseille France; ^5^ TAXON Estudios Ambientales S.L. Alcantarilla Spain; ^6^ CNRS, Délégation Provence et Corse Marseille France; ^7^ STARESO Marine Station Calvi, Corse France; ^8^ CEA Saclay—CNRS UMR12, Laboratoire Léon Brillouin Gif‐sur‐Yvette France; ^9^ National Museum of Natural History, Smithsonian Institution Washington DC USA; ^10^ ADENEKO Saint‐Girons France

**Keywords:** ARMS, biodiversity monitoring, eDNA, face‐by‐face sampling, marine benthic communities, Mediterranean Sea, metabarcoding

## Abstract

Monitoring marine biodiversity requires approaches capable of capturing its spatial and temporal complexity. DNA metabarcoding coupled with Autonomous Reef Monitoring Structures (ARMS) is increasingly used for this purpose, yet most applications still pool all sessile fractions and rarely benchmark molecular outputs against photographic observations. Here, we combined photographic analysis with cytochrome *c* oxidase I (COI) metabarcoding across 10 north‐western Mediterranean sites to compare and refine ARMS‐based monitoring protocols. We first optimized laboratory procedures (DNA extraction and polymerase choice) and applied the control‐driven, replicate‐aware VTAM pipeline to minimize false positives and ensure traceability. We then conducted the first face‐by‐face comparison of α‐ and β‐diversity between imaging and eDNA, metabarcoding each ARMS face separately rather than pooling samples. Metabarcoding detected ~15× higher site‐level richness and revealed stronger correlations with geographic distance and environmental gradients—which stemmed from its finer taxonomic resolution—whereas photography provided complementary information on macro‐taxa and surface cover. For metabarcoding, processing each face separately yielded much higher richness and stronger β‐diversity–distance correlations than with the NOAA pooling protocol, demonstrating that pooling inflates sampling variance, weakening ecological signal. Grouping the 17 faces into five structural categories offered a more operational alternative while further increasing α‐diversity and strengthening β‐diversity correlations. Overall, our results show that retaining ARMS microhabitat structure is critical for maximizing metabarcoding performance. Using five structural sessile fractions per ARMS combined with a control‐driven bioinformatic workflow provides a reproducible, scalable framework for long‐term eDNA monitoring and early detection of biodiversity change.

## 1 Introduction

Biodiversity protection is increasingly recognized as a global priority (Cowie et al. 2022). Global assessments warn that current biodiversity loss is unprecedented and threatens vital ecosystem services (Díaz et al. 2019a, 2019b). Marine ecosystems face intensifying pressures from climate change, ocean acidification, deoxygenation, overfishing, pollution and habitat destruction (Halpern et al. 2015; Duarte et al. 2020). Effective protection therefore depends on timely, evidence‐based monitoring of species composition and ecological change (Estes Jr et al. 2021; Miloslavich et al. 2018).

Meeting this need requires standardized, sensitive, and scalable methods that capture taxonomic and genetic diversity and their associated spatio‐temporal dynamics across broad geographic ranges (Laikre et al. 2020). For hard‐bottom benthic communities, two major challenges remain: (i) developing standardized sampling schemes and (ii) ensuring consistent, taxonomically reliable assessments (Danovaro et al. 2016, 2017). Historically, benthic assemblages were identified morphologically by taxonomic experts, which is a labour‐intensive process that depends on a diminishing pool of experts (Appeltans et al. 2012; Costello et al. 2013) and is prone to inconsistencies among experts, limiting reproducibility (Aylagas et al. 2016). These limitations hinder the long‐term and large‐scale monitoring plans required to track biodiversity change. The challenge is particularly acute in regions of exceptionally high species richness, such as the Mediterranean and many tropical seas, where small, morphologically similar or cryptic taxa dominate hard‐bottom assemblages (Coll et al. 2010; Bianchi et al. 2022; Obura 2012). In these biodiversity hotspots, the sheer number of species and the prevalence of cryptic or early‐life stages make comprehensive morphological inventories impractical.

Autonomous Reef Monitoring Structures (ARMS) offer a promising solution for standardized hard‐bottom sampling. Initially developed in tropical seas (Plaisance et al. 2011), they are now also deployed in temperate and polar environments (Couëdel et al. 2023; David et al. 2019; Obst et al. 2020; Pearman et al. 2018; Daraghmeh et al. 2025; Pagnier et al. 2025). ARMS provide a uniform, replicated habitat that becomes colonized by diverse sessile and mobile organisms. ARMS community composition has typically been characterized either through photography‐based analyses of (some of) the 17 exposed plate faces (David et al. 2019) or through DNA metabarcoding of the organisms scraped from the structure after retrieval (Leray and Knowlton 2015) and from their mobile fractions. The global ARMS programme provides a standardized protocol, from deployment to data analysis, particularly suitable for large‐scale biodiversity observatories such as ARMS‐MBON (Obst et al. 2020; Pearman et al. 2020). This programme integrates photography analyses and metabarcoding across European seas and polar regions, providing long‐term, FAIR (Findable, Accessible, Interoperable and Reusable) biodiversity data (Klymus et al. 2024; Takahashi et al. 2025) and delivering Essential Biodiversity Variables (EBVs; Miloslavich et al. 2018) as well as early detection of non‐indigenous species.

Both photography‐based and metabarcoding approaches have advantages for biodiversity assessment and can be partly automated thanks to advances in artificial intelligence and next‐generation sequencing, respectively (Beijbom et al. 2015; Pearman et al. 2018; Deiner et al. 2017; Taberlet et al. 2018). Furthermore, beyond community inventories, ARMS metabarcoding can also capture intraspecific genetic diversity and reveal cryptic population structure (Thomasdotter et al. 2023). On one hand, metabarcoding typically identifies taxa from bulk DNA extracts using universal primers and high‐throughput sequencing (e.g., Andújar et al. 2018; Brandt, Pradillon, et al. 2021; Pearman et al. 2020). However, false positives and false negatives can be generated without careful quality control (Zinger et al. 2019; Alberdi et al. 2018; Brandt, Trouche, et al. 2021). Here, we apply a stringent, control‐driven pipeline (VTAM; González et al. 2023) that minimizes such errors and ensures full traceability. On the other hand, photography‐based analyses can reach species‐level resolution, for some taxa, when performed by trained experts, but large monitoring networks often require coarser, higher‐taxonomic classifications (David et al. 2019; Pearman et al. 2018; Miloslavich et al. 2018).

The standard NOAA protocol for metabarcoding uses the two smallest mobile fractions (100–500 μm and 500–2000 μm) separately and a single composite sessile fraction scraped from all nine ARMS plates (Leray and Knowlton 2015; Pearman et al. 2020), thereby sacrificing microhabitat‐scale heterogeneity among individual plate faces as observed by David et al. (2019). To overcome this limitation and enhance ARMS‐based biodiversity assessments, we refined existing protocols through several methodological improvements. First, we evaluate laboratory procedures by comparing two DNA extraction kits and two polymerases to identify the most efficient combination for diverse marine assemblages. Second, we implement a face‐by‐face metabarcoding design in which the sessile community of each of the 17 colonizable ARMS faces is analysed individually rather than as a single pooled sample. This enables the first direct face‐by‐face comparison of hard‐bottom community composition using both photography‐based annotation and COI metabarcoding, providing unprecedented cross‐validation of α‐ and β‐diversity estimates. Third, we use two additional ARMS for each site where metabarcoding used pooled sessile fractions to test whether retaining face‐level resolution improves richness detection and strengthens correlations of community dissimilarity with environmental variables (geographic distance and environmental gradients) relative to the standard pooled NOAA protocol. Finally, we tested a more operational scheme in which five sessile fractions per ARMS (corresponding to distinct microhabitats) were analysed separately while including three ARMS units per site.

Here, we evaluate whether separating ARMS faces for metabarcoding improves biodiversity assessments relative to pooled designs. Specifically, we compare four monitoring protocols—one based on photography (images taken on the 17 individual plate faces of each ARMS) and three based on metabarcoding (full face‐by‐face processing of the same 17 faces, pooling all faces per ARMS and grouping faces into five structural categories)—to quantify their ability to recover α‐, β‐ and γ‐diversity and to detect geographic and environmental structuring of communities. Because ARMS provide contrasting microhabitats (upward‐ vs. downward‐facing surfaces, open vs. closed compartments, varying exposure to light), we hypothesized that retaining face‐level resolution would enhance the ecological signal recovered by metabarcoding. Accordingly, we predicted that: (i) metabarcoding of separated faces would reduce the impact of sampling variance and detect higher richness than pooled protocols, and (ii) geographic and environmental gradients would be more strongly recovered when ARMS faces are analysed individually or by structural category.

## 2 Material and Methods

### 2.1 Field Sampling and ARMS Sampling Design

**FIGURE 1:**
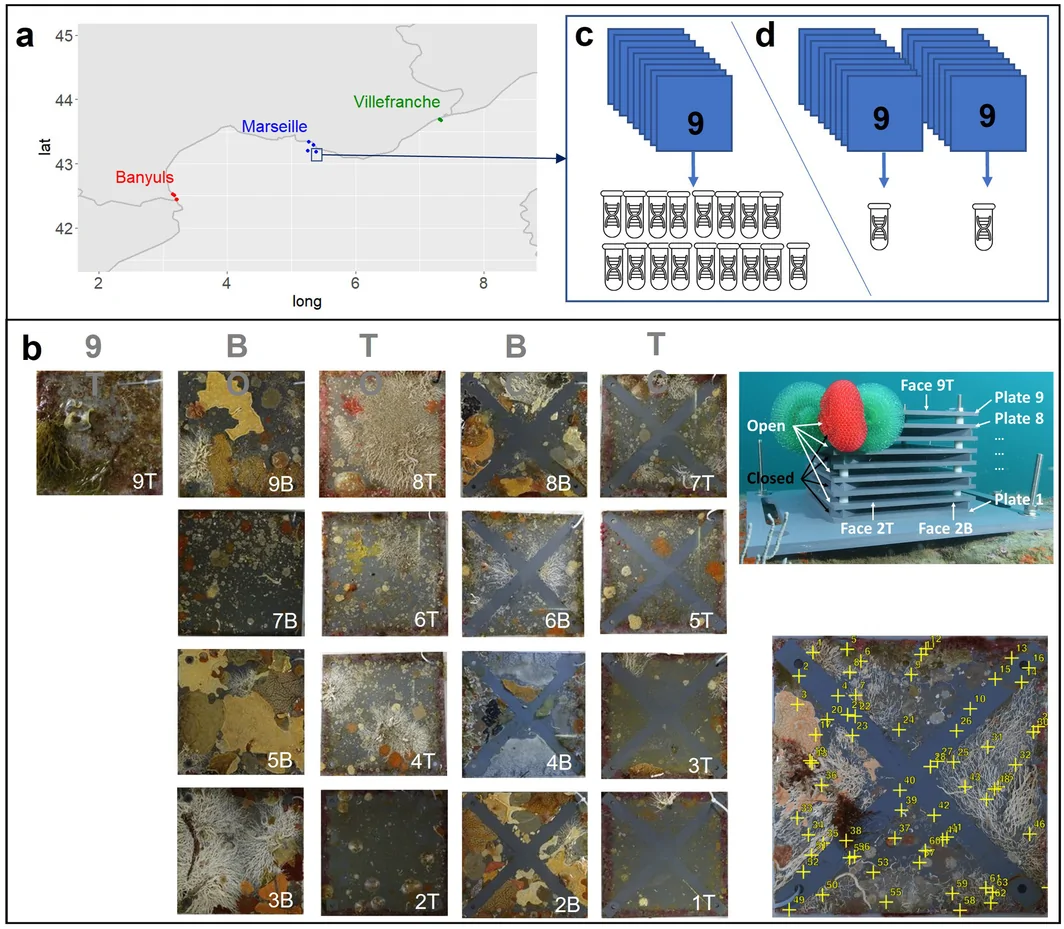
Study sites and experimental design for the 2018 dataset. The survey covered 12 sites distributed across three regions of the north‐western Mediterranean coast (France). Each site hosted three Autonomous Reef Monitoring Structures (ARMS), and each ARMS comprised nine plates providing 17 colonizable faces. (a) Map of the 12 study sites grouped by region. (b) Photography‐based analysis: Images of the 17 faces from a single ARMS, arranged in five columns representing the main structural face categories (9T, BO = bottom open, TO = top open, BC = bottom closed, TC = top closed). Bottom right, an example of random points overlaid on a photograph for taxonomic labelling by an expert assisted by artificial intelligence. (c–d) Metabarcoding design for one of the 10 sites where one ARMS was analysed face by face. (c) 17 separate sessile samples corresponding to the 17 faces, allowing direct comparison with the photographic data. (d) Pooled sessile fractions from each of the other two ARMS at the same site, processed as a single metabarcoding sample following the standard NOAA protocol. Only the 10 sites (out of 12) that followed the design illustrated in this figure and in Table 1 were used for comparative purposes.

Autonomous Reef Monitoring Structures (ARMS) were deployed by scuba divers at 12 sites distributed across three regions of the French Mediterranean coast (Figure 1) at depths of 16–22 m (Table 1). Units were immersed between 27 February and 7 April 2017 and retrieved between 5 March and 9 May 2018 (Table 1). Before dismantling and photographic documentation, each ARMS was kept in aerated seawater for 1 h to overnight. To remove vagile fauna, each plate was gently shaken prior to photography. After imaging, the sessile benthos was scraped from each face, blended for 15 s with sea water or ethanol, placed in a 40 μm mesh bag, slightly rinsed, then placed in a Falcon 50 mL tube filled with ethanol, and stored at +4°C until DNA extraction. For some samples processed in 2019, homogenization was performed after a period of storage in ethanol rather than immediately after collection. Detailed processing information for each sample is provided in the File S1 (MIEM sheet).

**TABLE 1:** Study sites and sampling details.

Site code	Site name	Region	Latitude	Longitude	Depth	Date in	Date out	Pooled ARMS	17 faces ARMS
BAN1	Port‐vendres S	Banyuls‐sur‐Mer	42.51327	3.13743	17 m	27/02/2017	17/04/2018	3	0
BAN2	Port‐vendres N	Banyuls‐sur‐Mer	42.52518	3.11050	17 m	28/02/2017	12/04/2018	2	1
BAN3	Cerbère N	Banyuls‐sur‐Mer	42.44818	3.17212	17 m	28/02/2017	19/03/2018	2	1
BAN4	Cerbère S	Banyuls‐sur‐Mer	42.44262	3.17405	17 m	01/03/2017	19/03/2018	2	1
MAR1	Veyron	Marseille	43.20683	5.25350	17.5 m	16/03/2017	07/05/2018	2	1
MAR2	Digue des catalans	Marseille	43.29167	5.34558	19 m	20/03/2017	05/03/2018	2	1
MAR3	Niolon	Marseille	43.34150	5.26525	16.5 m	03/04/2017	09/05/2018	2 (3)	1
MAR4	Plane	Marseille	43.19067	5.38233	17.5 m	07/04/2017	05/03/2018	2 (3)	1
VIL1	Pointe de la gavinette	Villefranche‐sur‐Mer	43.68683	7.32138	20 m	27/03/2017	13/03/2018	2	1
VIL2	Phare du cap ferrat	Villefranche‐sur‐Mer	43.67427	7.32803	18 m	28/03/2017	15/03/2018	2	1
VIL3	Grotte à corail	Villefranche‐sur‐Mer	43.68945	7.30883	17 m	28/03/2017	13/03/2018	3	0
VIL4	Cap ferrat est	Villefranche‐sur‐Mer	43.67675	7.33508	18 m	29/03/2017	14/03/2018	2	1

*Note:* For each site, the table reports depth, geographical coordinates (decimal latitude and longitude), dates of immersion and emersion, and the number of ARMS processed either as individual plate faces or as pooled sessile fractions for metabarcoding (numbers of samples are given in parentheses when duplicate analyses were performed on a single ARMS). Although most analyses in this study include only 10 sites (where 17 separate sessile community metabarcoding were carried out for one ARMS), we present all 12 sites for overall information on the SEAMoBB project.

Each ARMS consisted of nine superimposed plates (Figure 1), with an upper face (oriented upward) and a lower face (oriented toward the seafloor). In alternating inter‐plate spaces, crossbars subdivided the gap into four compartments (see Pearman et al. 2020). Plate faces were designated by plate number and orientation (e.g., 1T, 2B, 2T … 9T, where ‘T’ denotes the upward‐facing surface and ‘B’ the downward‐facing surface). For subsequent statistical analyses, we grouped the 17 faces into five structural categories:

- 9T, the uppermost face;
- BC (*bottom closed*): faces 2B, 4B, 6B, 8B;
- BO (*bottom open*): faces 3B, 5B, 7B, 9B;
- TC (*top closed*): faces 1T, 3T, 5T, 7T;
- TO (*top open*): faces 2T, 4T, 6T, 8T.

This hierarchical face‐type classification was used to test how fine‐scale microhabitat influences community composition, and to evaluate whether grouping faces by structural categories before DNA extraction is an efficient approach for metabarcoding‐based monitoring.

To assess the benefits of face‐by‐face sampling and compare imaging with metabarcoding at the finest level, we analysed at each site: (i) one ARMS unit with all 17 individual faces processed separately, and (ii) two ARMS units with all sessile material pooled into a single DNA extraction for each ARMS.

ARMS retrieved in 2018 were replaced by uncolonized ARMS during the same dive. This new set of ARMS was retrieved and dismantled in 2019 (Table S1). In the present study, these samples were exclusively analysed with metabarcoding under the new five structural categories scheme, with one DNA extraction and metabarcoding analysis for each category in each of the three ARMS per site.

### 2.2 Photo Analyses

We used a SONY DRC‐RX10M3 camera mounted on a fixed arm to photograph each face of every ARMS unit. Images were taken with a focal length of 35 mm, aperture F/6.3, exposure time 1/100 s and ISO 80 sensitivity. Photographs were captured under optimal natural light conditions. Each image was 4864 × 3648 pixels (350 ppi, 24‐bit colour depth, sRGB colour space). Images were subsequently cropped in *IrfanView* without quality loss and uploaded to the CORALNET website (https://coralnet.ucsd.edu/; Beijbom et al. 2015). Final cropped image files ranged from a few to about 10 MB, depending primarily on the proportion of uncolonized substrate.

Each image was then subdivided in 16 sub‐squares with 4 randomly drawn points, to a total of 64 points (stratified random sampling, Figure 1b). For the annotation process, a list of labels was prepared (i.e., names of taxa or names of non‐taxonomic categories to which a point could be assigned, fully described in File S1). During the annotation process, this list has been modified to add new taxa and to refine categories. For labels added to distinguish two categories which were previously merged in a single label, experts had to update already assigned images. The CORALNET search engine helped finding the corresponding images. Annotations were done manually until the automatic classifier was trained enough to propose labels, which were then either validated or corrected by experts (Beijbom et al. 2015; Chen et al. 2021; Williams et al. 2019). An initial model was generated after 50 annotated images, and a new model was trained every 50 additional annotated images. Over the course of 595 annotated images, 11 models were generated, with a maximum average accuracy of 69%. However, all label assignments were reviewed by experts, and the AI system served only as a tool to assist in the annotation process.

### 2.3 Metabarcoding Protocol: From Benchtop to Desktop

For DNA extraction, 1.5 mL of homogenate (see above: field sampling) was used. To minimize cross‐contamination, extractions were performed in individual tubes rather than in the 96‐well plate format of the extraction kit (see Corse et al. 2017). DNA extractions and COI metabarcoding followed the protocol described by Thomasdotter et al. (2023), with the following key steps: (i) DNA was extracted using the NucleoSpin Soil kit (Macherey‐Nagel, Germany); (ii) the cytochrome *c* oxidase subunit I (COI) gene was amplified in triplicate PCRs with primers IIICRrevN (3′‐GGNTGAACNGTNTAYCCNCC‐5′) and HBR2d (5′‐TAWACTTCDGGRTGNCCRAARAAYCA‐3′), which include a heterogeneity spacer and 11–13 nucleotide sample‐identifying tags, and target a broad spectrum of eukaryotic and algal phyla; (iii) a two‐step tailed PCR generated paired‐end, ready‐to‐load libraries; (iv) libraries were sequenced on an Illumina MiSeq using v2 chemistry (2 × 250 bp); and (v) a full series of negative and positive controls (two distinct mock communities) was incorporated.

Before the workflow was published in Thomasdotter et al. (2023), we conducted optimization trials (File S2) to assess how the choice of DNA extraction kit and polymerase affected sequencing yield and taxonomic breadth. Six ARMS‐derived samples (four sessile, two mobile) were each extracted at least twice using two DNA extraction kits, and all extracts were amplified in triplicate with two polymerases, generating 72 uniquely tagged amplicons for direct comparison. Optimization trials showed that the NucleoSpin Soil kit recovered broader diversity and that the Qiagen Multiplex polymerase produced higher read and ASV yields; this combination (described by Thomasdotter et al. 2023) was therefore adopted throughout the study. For bioinformatic processing we used VTAM v0.2.0 (Validation and Taxonomic Assignment of Metabarcoding data; González et al. 2023), a pipeline specifically designed to leverage the presence of technical replicates and control samples (including negative controls and mock communities). The core principle of VTAM is to optimize parameter values across multiple filtering steps to maximize the removal of false‐positive occurrences in control samples while retaining all expected variants (i.e., no false negatives in mock samples and the minimal possible number of false positives). Once optimized, these parameters are applied uniformly to all samples within the same sequencing library and run, under the rationale that parameter values validated on control samples are also appropriate for the corresponding experimental samples.

Briefly, paired‐end reads were merged, quality filtered, demultiplexed, and trimmed to remove sequencing tags and primers. Default parameters were used, except that the minimum and maximum lengths of merged and trimmed reads were set to 295 and 331 bp, respectively. Amplicon Sequence Variants (ASVs) were initially filtered to remove low‐frequency noise using low‐stringency default thresholds, excluding occurrences with low absolute or relative read counts within samples or across variants. Such occurrences likely originate from sequencing or PCR errors, tag‐jumps, or minor cross‐sample contamination. Optimal parameter values for these filters were then inferred from the control samples, after which the filtering procedure was repeated using the optimized parameters. Final parameter settings are provided in File S3.

To ensure reproducibility among technical replicates, only ASV occurrences detected in at least two of the three replicates per sample were retained, and replicates exhibiting composition profiles strongly divergent from the others were discarded. Subsequent filtering steps included the removal of potential chimeras and pseudogenes. Taxonomic assignment was performed in VTAM against the COInr database (version 2022‐05‐06; https://zenodo.org/records/6555985), after removing Insecta sequences to avoid spurious assignment of marine ASVs within Arthropoda (Meglécz 2023a). Assignments were made by the algorithm of mkLTG (Meglécz 2023b), using a stepwise identity threshold decreasing from 100% to 85%, ensuring high‐resolution identifications (genus or species) when close reference sequences were available and lower‐rank assignments when references were missing. The Minimum information for reporting of environmental metabarcoding data (MIEM checklist; Klymus et al. 2024) is reported in File S1.

### 2.4 Statistical Analyses of Diversity

#### 2.4.1 Assemblage Datasets

For the photography analyses, CORALNET output tables give, for each photography, the number of points assigned to each label. For most comparisons with metabarcoding, non‐biological labels such as *not colonizable* or *bare substrate* were removed, as was the label *unknown*, which generally represents undetermined organisms and could bias biodiversity estimates by conflating distinct taxa or by duplicating categories. For each remaining biological label, we calculated a standardized abundance by dividing the number of annotated points by the total number of labelled points on the image. From these data, we constructed three nested datasets: (i) PhoLab0, comprising all biological labels (38 in total); (ii) PhoLab2, in which several labels were merged to yield 34 broader biological categories; and (iii) PhoPhy, in which labels were pooled into nine higher‐level taxa corresponding to six animal phyla (Annelida, Bryozoa, Chordata, Cnidaria, Mollusca, Porifera), two photosynthetic groups (Ochrophyta and Rhodophyta) and a single label representing Foraminifera (File S1).

For metabarcoding, the filtered dataset was first analysed at the level of molecular operational taxonomic units (mOTUs)—clusters of amplicon sequence variants (ASVs) generated with Swarm v3 (Mahé et al. 2022) at *d* = 7. It was then aggregated into higher‐rank taxa (34 classes or 19 phyla or equivalent ranks) after removing mOTUs without any class or phylum‐level assignment (File S1) to enable comparisons with photographic data with more similar numbers of taxa. Taxonomic richness and relative abundance were calculated for each ARMS plate face and for both methods. Coverage‐based rarefaction and extrapolation curves were computed from incidence data (presence–absence across samples) with the package *iNEXT*, version 3.0.1 (Chao et al. 2014; Hsieh et al. 2016) for both photography and metabarcoding datasets. These curves estimate how diversity accumulates with sample number and provide asymptotic estimates of total richness. Taxonomic richness was expressed as the Hill numbers with parameter *q* = 0 (D_0_), the exponential of Shannon–Wiener entropy (Hill number with *q* = 1, D_1_), and the inverse of the Simpson concentration index (Hill number with *q* = 2, D_2_, i.e., the inverse of the sum of squared frequencies of each taxon).

#### 2.4.2 α‐Diversity

For each of the four datasets, α‐diversity (species richness) was quantified as the number of distinct photographic labels or mOTUs using PRIMER 7 (Clarke and Gorley 2015) with the PERMANOVA+ add‐on (Anderson et al. 2008). To verify that the choice of richness metric did not affect cross‐method comparisons, we also calculated Shannon and Simpson indices and the related (not identical) Hill numbers (*q* = 1 and *q* = 2, respectively); because these metrics were strongly correlated with sample richness, they were not discussed further. Concordance between methods was evaluated by computing Pearson and Spearman correlation coefficients for paired photographic and metabarcoding α‐diversity values from the same plate‐face samples. Relative‐abundance barplots were generated with the *ggplot2* package, version 3.5.2 (Wickham et al. 2016) using all photographic labels and metabarcoding mOTUs aggregated to higher‐rank taxa. Linear regressions confirmed that correlations were consistent across the different diversity indices (not shown). Correlation matrices and visualizations were generated with the *corrplot* package, version 0.95 (Wei et al. 2017). These analyses were performed at two spatial scales: the sample level (166 individual plate faces) and the site level (10 sites represented by the summed abundances of the 17 faces of their analysed ARMS).

#### 2.4.3 β‐Diversity, Geography and Environment

For each dataset, β‐diversity was assessed from pairwise community dissimilarities calculated with the Bray–Curtis index (after a square root transformation of standardized abundances) and, for comparison, the Sørensen index, which relies solely on presence–absence data and is sometimes preferred for metabarcoding as read numbers may imperfectly reflect biomass or abundance (Deiner et al. 2017; Hajibabaei et al. 2019). Mantel tests were used to evaluate correlations in community dissimilarity between photographic and metabarcoding data (using the package ape, version 5.8.1; Paradis et al. 2019) at both sample level (all individual plate faces) and site level (45 non‐redundant site pairs). To examine spatial and environmental structuring, least‐cost sea distances (km) were computed among sites and correlated with Bray–Curtis dissimilarities using Mantel tests.

Environmental variables used in this study were derived from E.U. Copernicus Marine Service Information products. Physical time series (1987–2019) were obtained from the MEDSEA_MULTIYEAR_PHY_006_004 reanalysis (Escudier et al. 2020), and biogeochemical time series (1999–2018) from the MEDSEA_ANALYSIS_FORECAST_BIO_006_008 reanalysis (Teruzzi et al. 2021). For the 12 ARMS anchor points (latitude, longitude), 15 variables were extracted from 180 monthly CSV files, including temperature (thetao, bottomT), salinity (so), eastward and northward current velocity (uo, vo), sea surface height (zos), mixed layer thickness (mlost), nitrate (nit), phosphate (pho), phytoplankton carbon biomass (pcb), chlorophyll (chl), primary production (ppn), dissolved oxygen (dox), partial pressure of CO_2_ (pco) and pH (ph). For each anchor point, basic statistics (mean, median, maximum, minimum, range, and first and third quartiles) were calculated for all variables using data averaged vertically between 1 and 50 m, on 4 × 4 km grids, from the full temporal coverage of the physical (1987–2019) and biogeochemical (1999–2018) models. All computations were performed in GRASS GIS version 8 (GRASS Development Team et al. 2025).

Euclidean distances based on scaled environmental variables (Latitude, Longitude, mean_thetao, range_thetao, mean_bottomT, range_bottomT, min_BottomT, max_bottomT, so, uo, vo, zos, mlost, nit, pho, pcb, chl, ppn, dox, pco, ph) were used in Mantel tests and partial Mantel tests (i.e., after sea least cost distance was accounted for) to disentangle geographic and environmental effects. Permutational multivariate analyses of variance (PERMANOVA; PRIMER 7 with PERMANOVA+) were performed on all photographic and metabarcoding datasets (across three taxonomic aggregation levels each) with models including combinations of factors such as region, site (nested within region or alone), face type (9T, BO, BC, TO, TC), defined by the combination of orientation (top vs. bottom), and compartmentalization (open vs. closed) (Table 2). We tested for homogeneity of dispersion in community composition across site, face type and region using the PERMDISP routine in PRIMER7 PERMANOVA+. For metabarcoding data only, we compared the ARMS units analysed face‐by‐face (one per site) against the two companion ARMS per site whose sessile fractions were pooled by means of barplots of relative abundance and richness by phylum and region. We also regressed site‐level β‐diversity with the logarithm of ‘geographic distance + 1’ and with Euclidean environmental distances using Mantel tests as above. R analyses used the version 4.2.0 (R Core Development Team 2023) and all scripts and input files are in the github repository indicated in the data availability statement.

**TABLE 2:** Main effects from PERMANOVA models.

Design	Factors	Metabarcoding			Photography		
		1493 mOTUs	34 classes	19 phyla‐like	38 labels	34 lab2	10 phyla
1	Region	***	***	***	***	***	***
	Face type	***	***	***	***	***	***
2	Region	**	ns	ns	*	*p* = 0.0503	*
	Site (Region)	***	***	***	***	***	***
	Face type	***	***	***	***	***	***
3	Region	***	***	***	***	***	***
	Orientation	***	***	***	***	***	***
	Compartmentation	*	**	*	***	***	***
4	Site	***	***	***	***	***	***
	Orientation	***	***	***	***	***	***
	Compartmentation	***	*	***	***	***	***

*Note:* Summary of the primary factors explaining variation in community composition, as identified by permutational multivariate analysis of variance (PERMANOVA). The table reports the main effects retained in the final models for both photographic and metabarcoding datasets, with associated test statistics and significance levels using symbols (***p < 0.001, **p < 0.01, *p < 0.05), unless specified.

## 3 Results

### 3.1 Taxonomic Composition, Rarefaction Patterns and Face‐Type Contrasts

Photography and metabarcoding revealed distinct yet partially overlapping characterization of ARMS sessile communities. To ensure robust cross‐method comparisons, we removed four low‐diversity samples—due to one photographic sample lacking any biological label (BAN3‐3_T5) and three metabarcoding samples with very few reads and mOTUs (BAN2‐3_T9, BAN2‐3_B8 and VIL2‐1_B8). In the photographic dataset, non‐biological categories dominated: ‘bare substrate’ accounted for ~43% of the surface of all 598 images, ‘not colonizable’ for ~12% (reflecting ARMS structural elements such as compartments and screws) and ‘detritus’ for ~5%. Comparisons were therefore restricted to the 38 biological labels (algae, animals and Foraminifera) distributed across nine to 10 phyla (File S1).

Metabarcoding of the remaining 166 paired samples detected 1493 mOTUs, an order of magnitude higher than the 38 photographic labels. Out of these ~1500 mOTUs, 150 were assigned to 139 species, some also observed visually. Twenty‐six of the 38 labels found in the 166 photographs were species (13) or genus (13) names. Only two of them were identified from the 166 metabarcoding samples (the bryozoans *Idmidronea atlantica* , *Schizomavella mamillata*) but seven additional shared species (metazoans and algae) were found when considering all SEAMoBB samples. Some appeared or became identifiable visually only in ARMS immersed for longer durations; others were only identified via close‐ups or a hand lens. Eight species ( *Amphipholis squamata* , *Antedon bifida* , *Bittium latreillii*, *B. reticulatum* , *Limaria hians* , *Mimachlamys varia*, *Paracentrotus lividus* and *Pseudoceros velutinus* ) detected via metabarcoding (from sessile fractions) had been visually identified but excluded from the photography dataset, as they were considered part of the mobile fraction.

Of the 12 species identified in the 166 photographs but absent from the metabarcoding, two had been detected in other SeAMoBB French ARMS metabarcoding samples ( *Caberea boryi* and *Ciona intestinalis* ) and one, *Anomia ephippium* —known for COI primer site polymorphism (Chenuil, pers. comm.)—had all specimens removed for population genetics. Of the nine remaining species, four lacked COInr entries ( *Miniacina miniacea* , *Platonea stoechas*, *Schizobrachiella sanguinea*, *Tubulipora plumosa* ). Two had entries but were assigned at higher ranks because: (i) the barcode sets of two species in COInr (*Ciona roulei* and *Ciona intestinalis* ) were too similar, so some variants were assigned only at the genus rank, or (ii) a mislabelled COInr sequence caused Alcyonium coralloides to be assigned at the Eumetazoa rank. These deductions came from reassigning mOTUs against a COInr subset of the 12 ‘missing’ species. Therefore, three species, *Ascandra contorta*, *Ascidia mentula* and *Ascidiella scabra* were likely affected by amplification failure.

**FIGURE 2:**
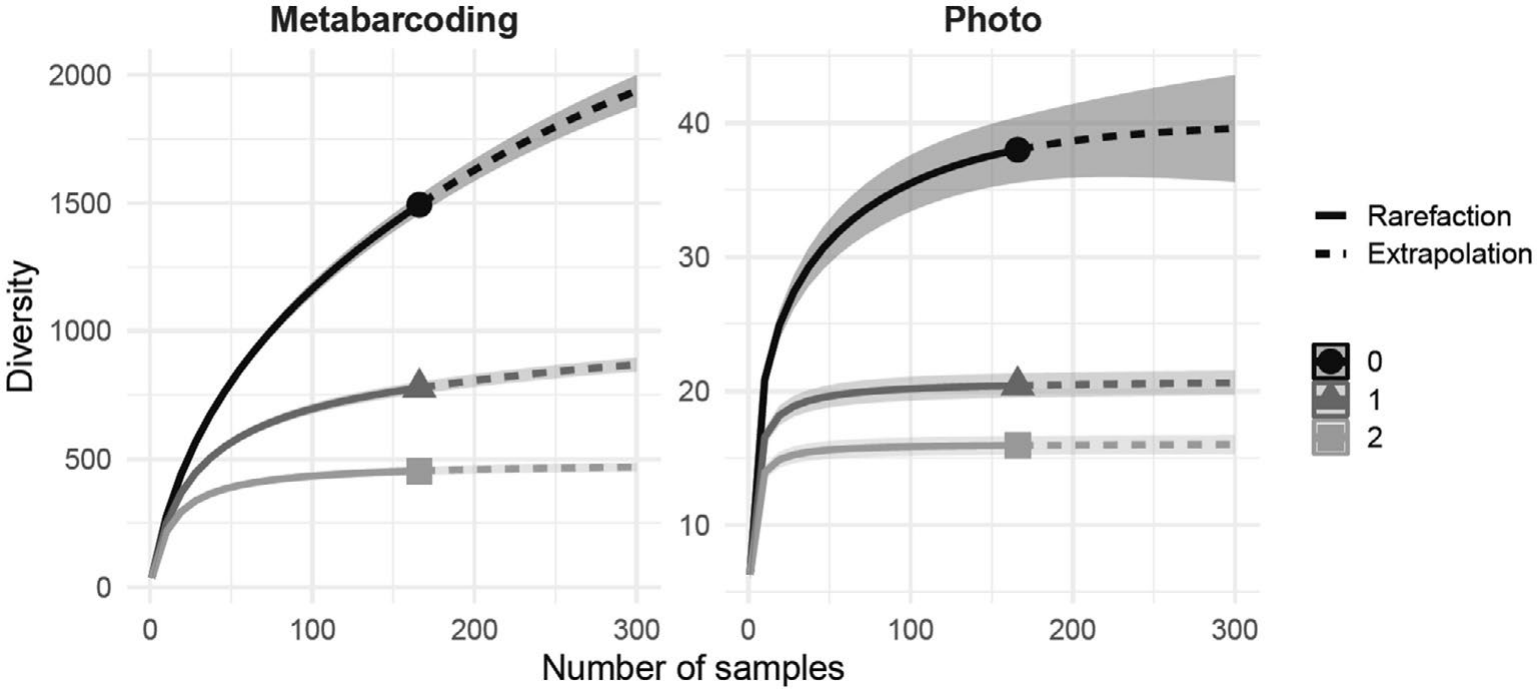
Taxa rarefaction and extrapolation curves. The number of metabarcoding molecular operational taxonomic units (mOTUs) or photographic taxonomic labels is plotted on the vertical axis as a function of the number of photographic or metabarcoding samples on the horizontal axis. Curves represent three types of Hill numbers defined by the *q* parameter: Taxonomic richness (*q* = 0), i.e., the number of labels or mOTUs (black); Shannon‐like diversity (*q* = 1), i.e., the exponential of Shannon entropy (grey); and Simpson‐like diversity (*q* = 2), i.e., the inverse of Simpson concentration (light grey). Shaded areas denote standard‐error confidence intervals. The metabarcoding dataset here corresponds to the 17‐faces design (for comparisons with photography).

Sample richness ranged from 1 to 13 biological labels for photography and from 7 to 76 mOTUs for metabarcoding, underscoring the much finer taxonomic resolution of DNA data. Coverage‐based rarefaction and extrapolation (Figure 2) confirmed this pattern: photographic richness approached saturation, whereas metabarcoding curves remained far from asymptotic, especially for *q* = 0 (rare taxa). Asymptotic Hill numbers of order 0 (richness), order 1 (abundant taxa, related to Shannon's entropy) and order 2 (dominant taxa, related to Simpson's index) were 40 (s.e. = 6.9), 20.4 (s.e. = 0.5) and 16 (s.e. = 0.44) labels for photography, compared with 2391 (s.e. = 75), 902 (s.e. = 16) and 455 (s.e. = 10) mOTUs for metabarcoding.

**FIGURE 3:**
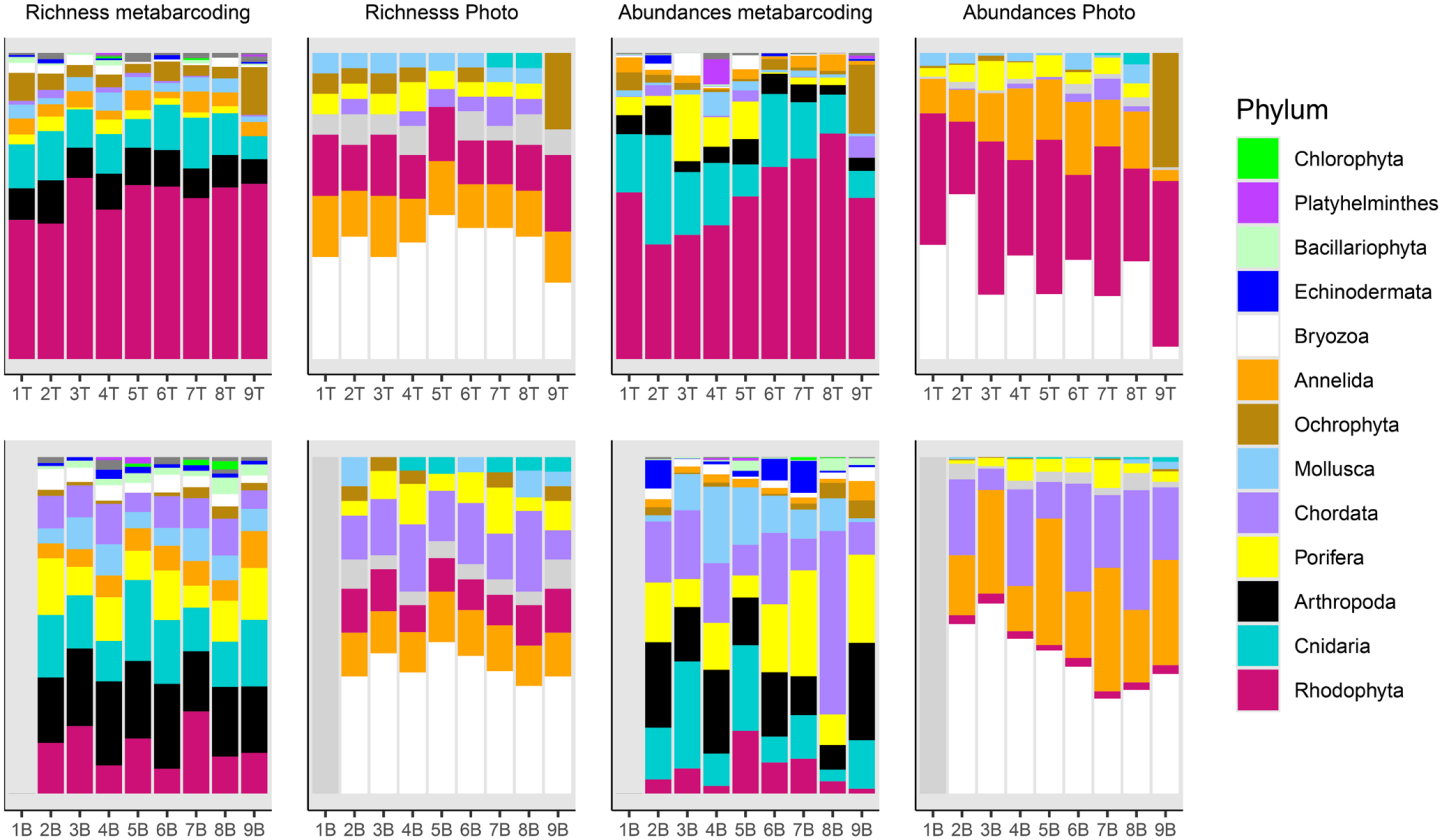
Barplots of taxonomic composition by phylum for each method and plate face. Top (1T to 9T) and bottom plate faces (2B to 9B) are shown in the upper and lower panels, respectively. Relative richness (left panels) and relative abundance (right panels) are presented for metabarcoding and photography‐based analysis. Note that Chordata consist mainly of Ascidiacea rather than fishes, which were absent from the photographic data and relatively rare in the metabarcoding results. Unassigned mOTUs are not displayed, and all mOTUs belonging to sparsely represented phyla or higher‐rank taxa are pooled in dark grey, a category not shown separately in the legend. The taxa in the unique legend are ordered by their abundances in metabarcoding. The metabarcoding dataset used here corresponds to the 17‐faces design (for comparisons with photography).

Taxonomic distributions also differed between methods and among plate faces (Figure 3). Both approaches revealed higher Rhodophyta abundance on top faces, but Bryozoa and Annelida were proportionally far richer in photographs, whereas Arthropoda and Echinodermata were detected only by metabarcoding.

**FIGURE 4:**
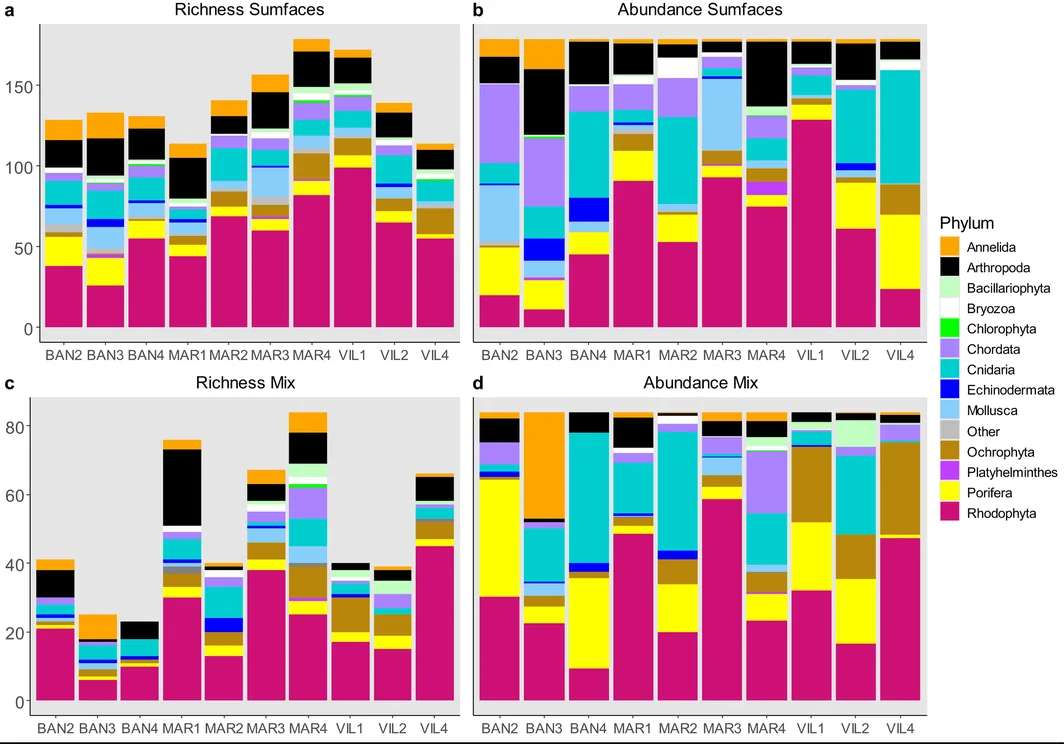
Comparison of metabarcoding designs: separate versus pooled ARMS faces. Bar plots show, for each site, the richness (in mOTUs) (a, c) or relative abundance (b, d) by phylum per site. Top panels (a, b): Each bar represents the combined total of 17 individual face samples from a single ARMS (with minor exceptions where some faces were discarded; see text). Bottom panels (c, d): Each bar represents the combined total of two pooled sessile‐fraction samples, each derived from a whole ARMS, following the standard NOAA protocol. This dataset corresponds to the ARMS retrieved in 2018 only. Note the different vertical scales between panels (a) and (c).

Pooling all sessile fractions per ARMS drastically reduced diversity in metabarcoding: 22 pooled samples (two ARMS per site plus one biological replicate for MAR3 and MAR4) yielded 559 mOTUs—less than half of the 1493 mOTUs obtained from 166 separated‐face samples from 10 ARMS (Figure 4). Phylum abundances were more even in the face‐by‐face design, whereas pooled samples over‐represented Ochrophyta and, to a lesser extent, Porifera (Figure 4).

In the 2019 metabarcoding dataset, based on five structural face categories across three ARMS per site, 137 samples were retained after quality filtering (13 removed from 150). These yielded 1541 distinct mOTUs, slightly exceeding the 1493 recovered from the 166 individual‐face samples analysed in 2018.

### 3.2 Relationships Between Photographic and Metabarcoding Diversity

**FIGURE 5:**
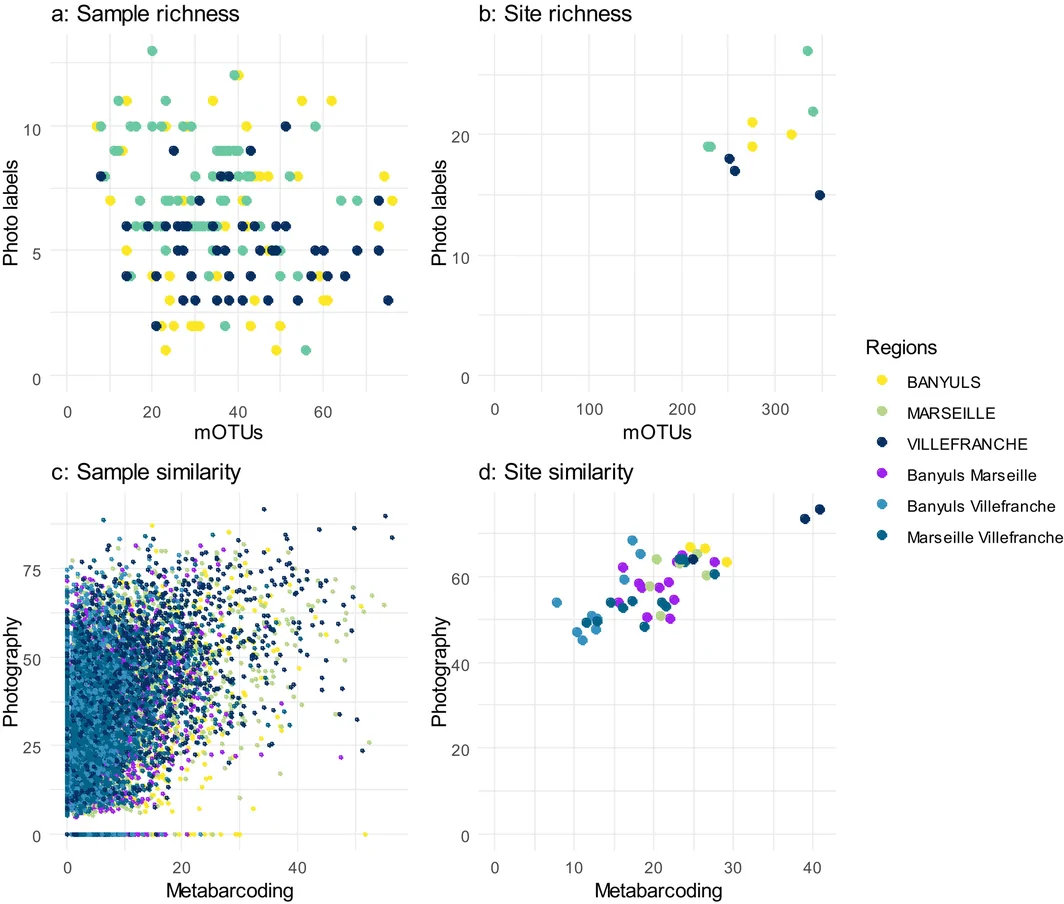
Correlation of diversity measures between photographic and metabarcoding methods (lowest taxonomic ranks). Horizontal axes show values derived from metabarcoding data (1493 mOTUs), and vertical axes show values derived from photographic data (38 labels, labelset 0). Upper panels (a, b) present α‐diversity (richness), and lower panels (c, d) present β‐diversity (Bray–Curtis similarity) for pairwise comparisons. Left panels (a, c) display sample‐level data, whereas right panels (b, d) display site‐level data. The metabarcoding dataset used here corresponds to the 17‐faces design (for comparisons with photography).

**FIGURE 6:**
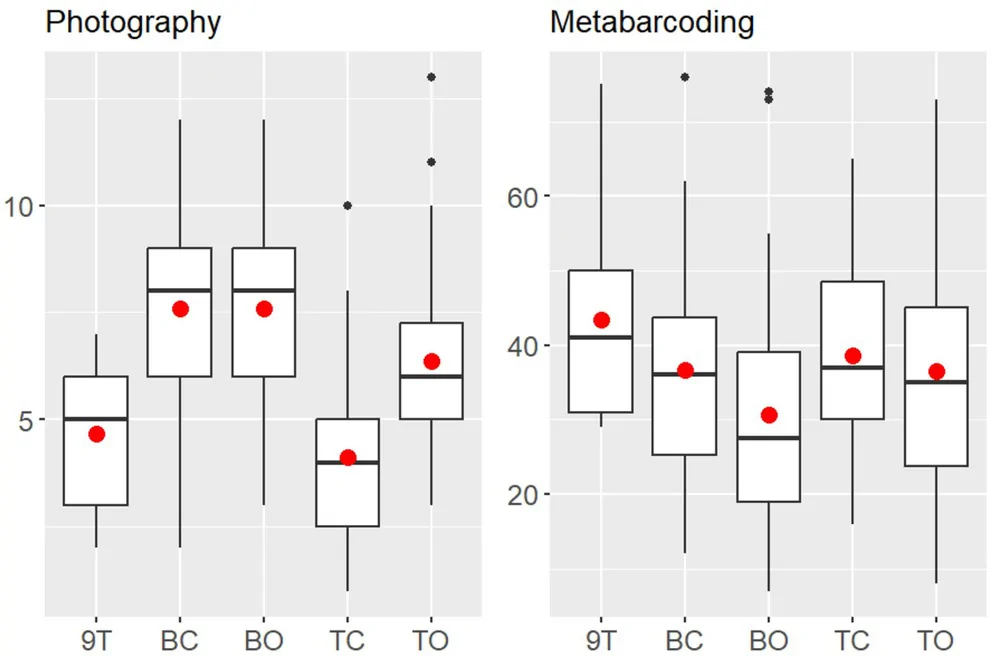
Taxonomic richness across five ARMS structural face categories and both survey methods. Left panel: photography‐based analysis (vertical axis: number of distinct biological labels). Right panel: metabarcoding (vertical axis: number of molecular operational taxonomic units, mOTUs). Red dots indicate the mean; thick horizontal bars show the median; box limits correspond to the first (Q1) and third (Q3) quartiles; whiskers extend to the most extreme data points within 1.5 × IQR (interquartile range); and isolated points represent outliers beyond this range. Structural face categories that are least diverse in photographic analyses (9T, TC) tend to be the most diverse in COI metabarcoding. The metabarcoding dataset used here corresponds to the 17‐faces design (for comparisons with photography).

Sample‐level α‐diversity values showed a slight but significant negative correlation between photographic and metabarcoding datasets (Pearson *R* = −0.17, *p* = 0.0245; Figure 5). After pooling all plate‐face samples within each site, the relationship became positive but non‐significant (*R* = 0.24, *p* = 0.51), insignificance being likely driven by an outlier site from the Villefranche‐sur‐Mer region. Site richness was roughly 15‐fold higher for metabarcoding than for photography (Figure 5). When taxa were aggregated to higher ranks, positive site‐level correlations became clearer. The strongest correspondence (Figure S1) occurred when metabarcoding data collapsed to 34 classes were compared either with the full photographic label set (38 labels; *R* = 0.71, *p* = 0.021), or with the intermediate labelset 2 (34 labels; *R* = 0.69, *p* = 0.026; Figure S1). In contrast, β‐diversity (Bray–Curtis dissimilarity) exhibited consistently positive and stronger correlations between methods, both at the sample level (*R* = 0.39, Mantel *p* = 0.0001; Figure 5) and at the site level (*R* = 0.72, Mantel *p* < 0.0001; Figure S1). These positive correlations persisted at all taxonomic aggregation levels, but coefficients declined steadily with coarser taxonomic ranks, reaching non‐significant values (*R* ≈ 0.20) when both datasets were reduced to phylum level (Figure S1). The contrasting signs of α‐diversity correlations at sample versus site level can largely be attributed to strong differences among ARMS face types: for instance, the superior 9T face was the most species‐rich in metabarcoding but among the least diverse in photographic data, whereas bottom‐open (BO) faces showed the opposite trend (Figure 6).

### 3.3 Geographical and Ecological Effects on β‐Diversity

**FIGURE 7:**
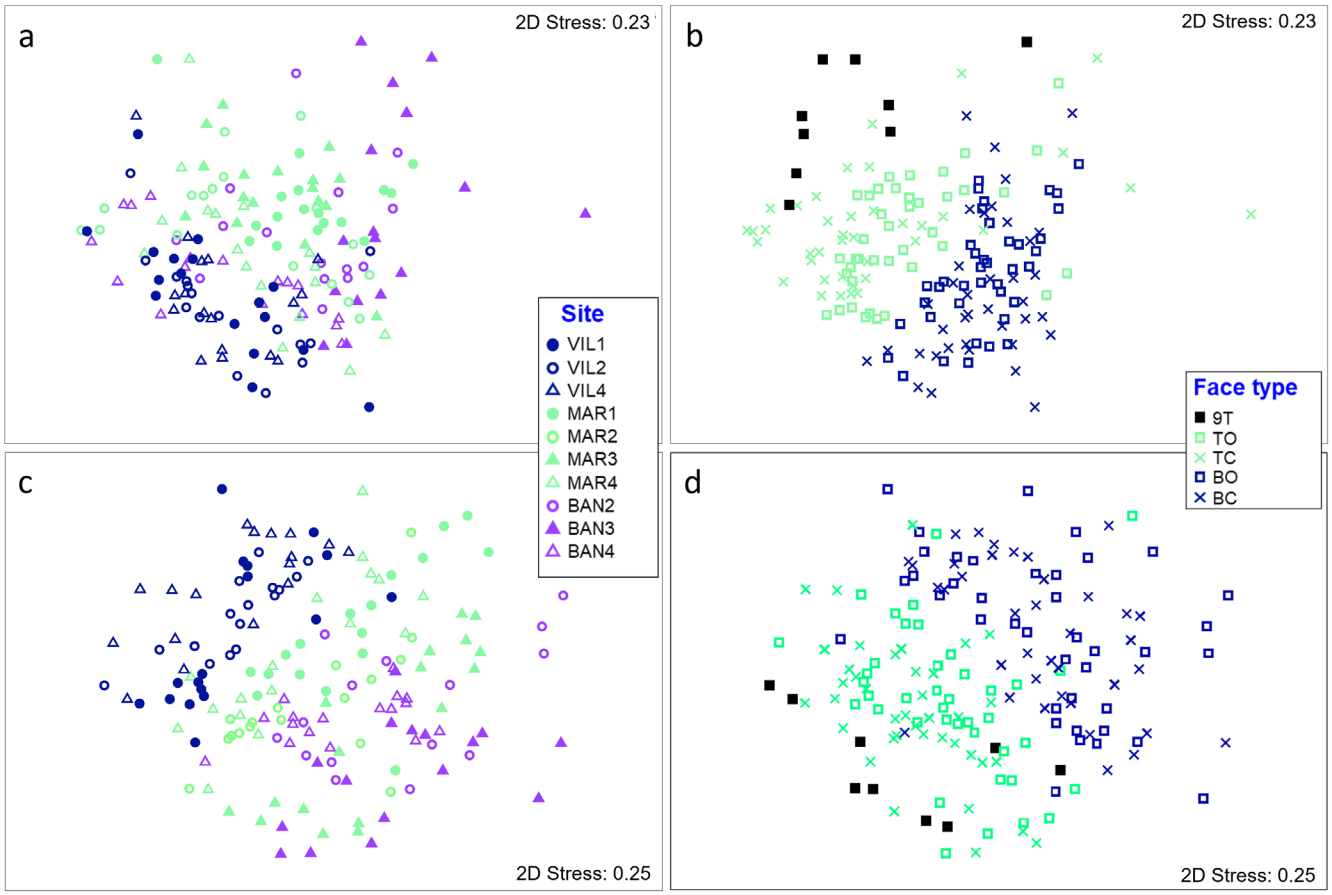
β‐diversity comparison using non‐metric multidimensional scaling (NMDS). Ordination of samples based on Bray–Curtis dissimilarity computed from standardized and square‐root–transformed abundances (read numbers for metabarcoding; surface cover for photography). Top panels (a, b): photography data (38 labels, labelset 0). Bottom panels (c, d): metabarcoding data (1493 mOTUs). Left subpanels (a, c): Points coloured by site and by region. For visibility (and trying to account for colour blindness) we changed the colour representing the region of Banyuls from yellow (in Figures 2 and 4) to violet. Right subpanels (b; d): Points coloured by face type, with symbols indicating compartmentation (× = closed faces; open squares = open faces) and orientation. The metabarcoding dataset used here corresponds to the 17‐faces design (for comparisons with photography).

Both photographic and metabarcoding datasets revealed strong spatial and ecological structuring of community composition. PERMDISP detected differences of dispersion among regions (*p* = 0.0001, *p* = 0.0006 for metabarcoding and photography respectively), sites (*p* = 0.0256 and *p* = 0.0001) and face types (*p* = 0.0165 for metabarcoding only). PERMANOVA models (Table 2) detected highly significant effects of region, site and plate‐face type on β‐diversity. When region and site (nested within region) were included jointly, regional effects weakened (higher *p*‐values), indicating that much of the regional signal was captured at the site level. Face orientation (top vs. bottom) was consistently highly significant, whereas compartmentation (open vs. closed) was strongly significant for photographic data and moderately significant for metabarcoding. Non‐metric multidimensional scaling (nMDS) ordinations (Figure 7) corroborated these patterns: samples separated clearly by face orientation, open–closed differences were more diffuse, and regional structure appeared as an east–west gradient along a diagonal axis. This geographical gradient was slightly more pronounced for metabarcoding, whereas fine‐scale ecological differentiation within‐ARMS (related to light and microhabitat) was clearer in the photographic ordination. Although dispersion in community composition varies between regions, sites or face types, nMDS plots confirm that PERMANOVA results reflect actual shifts in community composition, not just differences in dispersion. Analyses based on the Sørensen index, which is based on presence–absence rather than abundance, yielded very similar results (data not shown).

**FIGURE 8:**
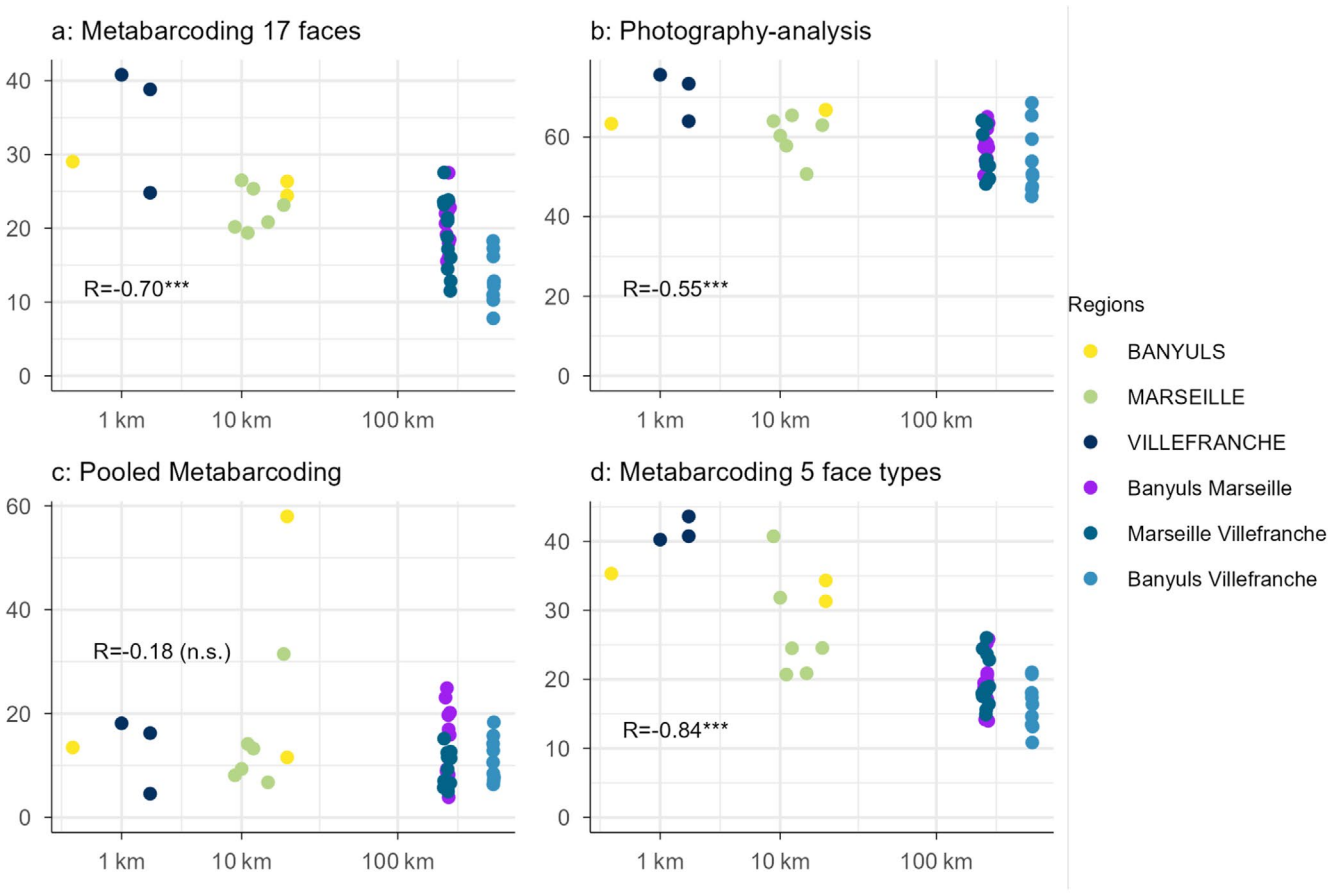
Community similarity versus geographic distance. Scatterplots of Bray–Curtis similarity (vertical axis) against the least‐cost geographic distance (km) (horizontal axis, logarithmic scale). Metabarcoding data—(a) separate faces (166 samples before pooling by site, retrieved in 2018), (c) pooled sessile fractions (22 samples before pooling by site, retrieved in 2018) and (d) five sessile fractions (137 samples before pooling by site, ARMS retrieved in 2019). Right panel (b): Photography‐based data (2018). Pearson correlation coefficients and significance levels are indicated on each plot. The vertical axis scale varies among panels.

Community similarity declined significantly with the logarithm of sea least‐cost geographic distance for both methods, but more tightly for metabarcoding (Mantel *R* = 0.70, *p* = 0.00003) than for photography (*R* = 0.55, *p* = 0.00038; Figure 8a,b). The weaker relationship for photography likely reflects unusually high similarity between the most distant regions (Banyuls‐sur‐Mer and Villefranche‐sur‐Mer) and low similarity among some Marseille sites. Taxonomic aggregation reduced distance–decay correlations for both datasets, though correlations remained significant. When metabarcoding mOTUs were collapsed to class or phylum, Mantel coefficients decreased to *R* = 0.43 (*p* = 0.00183) and *R* = 0.34 (*p* = 0.00482), respectively (File S5). Similarly, aggregating photographic data to labelset 2 or phylum yielded *R* = 0.47 (*p* = 0.00138) and *R* = 0.27 (*p* = 0.01593) (File S5). By contrast, pooled‐faces metabarcoding performed worst, producing the lowest and non‐significant correlations with geographic distance (*R* = 0.18, *p* = 0.1359; Figure 8c). Metabarcoding based on five structural face categories (three ARMS per site, 2019 dataset) yielded the strongest distance–decay relationship (*R* = 0.84, *p* = 0.00002; Figure 8d), outperforming the 17‐face design despite using fewer samples (137 vs. 166). Regression intercepts indicated that similarity at 0 km averaged 64% for photography, 27% for face‐by‐face metabarcoding and only 16% for pooled metabarcoding, highlighting the reduced consistency of pooled samples. The greater distance of metabarcoding rarefaction curves from their asymptotes likely contributes to this pattern, indicating higher sampling variance relative to photography.

Environmental variables also helped explain β‐diversity patterns (File S4). Mantel tests showed that scaled Euclidean distances in several environmental descriptors were significantly correlated with community dissimilarity, although geographic distance consistently explained more variance. Partial Mantel tests controlling for geography identified four significant predictors of species composition for metabarcoding (eastward current velocity, nitrate, phosphate and primary production) and two for photography (nitrate and chlorophyll concentration). In addition, chlorophyll was nearly significant for metabarcoding, and eastward current velocity, phosphate, phytoplankton carbon biomass, and primary production approached significance for photography. Metabarcoding using five structural face categories (three ARMS per site) identified six significant environmental variables, the highest number across all datasets.

## 4 Discussion

Our key methodological advance—separating ARMS faces for metabarcoding—proved decisive for improving biodiversity assessments. By increasing detected richness, reducing sampling variance, and strengthening biogeographic and environmental correlations, this approach markedly outperformed the standard NOAA pooling protocol and provides a new benchmark for standardized eDNA monitoring. In the following discussion, we examine (i) the complementarity between photography and metabarcoding, (ii) the causes of their differing performance and (iii) the implications of our results for sampling design and for routine monitoring frameworks—with a focus on the benefits of separating ARMS structural face categories (or all 17 faces) compared with pooled designs for metabarcoding. Finally, we consider the broader implications of these findings for long‐term marine biodiversity monitoring and management.

### 4.1 Complementarity of Photography and Metabarcoding

α‐ and γ‐diversity were markedly higher in metabarcoding than in photographic analyses, even under a stringent bioinformatic pipeline (González et al. 2023). This pattern reflects the finer taxonomic resolution of COI metabarcoding—which can distinguish cryptic species lacking morphological differentiation (Cahill et al. 2024; Chenuil et al. 2019)—as well as its ability to detect a broader size range of organisms. The two methods also differed in how they resolved variations among ARMS structural face categories. Photography recorded the lowest diversity on superior faces (9T), whereas these yielded the highest richness by metabarcoding. A likely explanation is that dense macroalgal cover (e.g., Ochrophyta such as *Dictyota* spp.) obscures underlying microhabitats from visual detection, while providing more colonizable surface for cryptic taxa that are well captured by DNA. Conversely, photography revealed the lowest richness on top‐closed faces (a pattern not observed in metabarcoding) likely reflecting differences in light exposure and the availability of bare‐substrate. These contrasting face‐type effects explain the negative correlation of α‐diversity between methods at the sample level, whereas pooled site‐level richness showed a positive—but non‐significant—correlation, possibly constrained by the limited number of sites (*n* = 10).

The taxonomic spectra recovered by the two methods overlapped but differed in detail. Every phylum detected by photography was also identified via metabarcoding. However, groups like Bryozoa and Annelida were abundant in photographs but rare in metabarcoding, unlike Cnidarians. Arthropods, absent in photographs, were abundant in metabarcoding, likely due to poor separation of sessile and mobile compartments and the high sensitivity of PCR (Pearman et al. 2020).

Of the nine species identified in photographs but never detected in metabarcoding, three likely resulted from PCR failure or inefficiency, four from missing entries in the reference database, one from a database error, and one from a barcode marker resolution issue. Many photography–metabarcoding discrepancies could therefore be reduced through improved reference databases and taxonomic curation, whereas others reflect intrinsic limitations of single‐marker metabarcoding. Metabarcoding's failure to detect a taxon may also result from low DNA abundance or stochastic sampling effects (Lamb et al. 2019; van der Loos and Nijland 2021). Most metabarcoding diversity nevertheless consisted of mOTUs lacking phylum‐level assignments, either because reference sequences remain unavailable for many lineages or because the amplified COI fragments originated from poorly characterized non‐metazoan organisms, including unicellular eukaryotes and even prokaryotes (Collins et al. 2019; Hintikka et al. 2022).

Although photographic analysis detected far fewer taxa than metabarcoding, both approaches effectively revealed significant effects of all tested spatial and ecological factors on community composition. These factors included region (three regions separated by up to ~395 km), site within region (0–20 km apart), and ARMS face type (9T, top open, top closed, bottom open, and bottom closed). However, subtle but consistent differences emerged in the drivers of community composition detected by each method; *p*‐values differed slightly between them, indicating nuanced contrasts in sensitivity. Photography detected more significantly than metabarcoding differences in microhabitat within ARMS (plate compartmentation). By contrast, metabarcoding appeared more tightly correlated with geographical distance and environmental variables (see below).

Together, these comparisons show that photography and metabarcoding provide complementary insights into marine hard‐bottom biodiversity. Photography offers direct visual estimates of macroscopic cover (e.g., macroalgae), while metabarcoding captures cryptic, microscopic or early‐stage organisms and preserves fine‐scale heterogeneity. Future monitoring programmes may combine both approaches according to management objectives: photography for dominant taxa and surface cover, and metabarcoding for cryptic diversity, early colonizers and compositional change in turbid, complex or species‐rich environments. The broad convergence between methods supports their reliability and robustness, and the stronger geographic and environmental signal in metabarcoding highlights its greater sensitivity to subtle compositional change.

### 4.2 Taxonomic Resolution Explains the High Sensitivity of Metabarcoding to Environmental and Spatial Gradients

In contrast with α‐diversity patterns at sample levels, β‐diversity patterns were strongly congruent between methods, with significant Mantel correlations at both sample and site levels (Figure 5, Figure S1). This indicates that both approaches reliably captured community structure. However, metabarcoding revealed stronger correlation of community similarity with geographic distance (*R* = 0.70 vs. 0.55 for photography, with a single ARMS per site). Likewise, both methods revealed similar environmental variables correlated with community dissimilarity, but metabarcoding consistently produced higher statistical power, with several variables that were marginally non‐significant in photographic data becoming significant in metabarcoding. Aggregating mOTUs into higher ranks (i.e., classes and phyla) progressively weakened these correlations, ultimately falling below those from photography, demonstrating that the stronger biogeographic and environmental signal with metabarcoding stems from its finer taxonomic resolution.

We expected mOTU‐level metabarcoding to reflect geographic distances more clearly than photography because it captures intraspecific diversity (e.g., Thomasdotter et al. 2023; Turon et al. 2020) that is frequently structured by isolation‐by‐distance or oceanographic connectivity (Legrand et al. 2022). Marine species frequently exhibit spatial variation in mitochondrial COI haplotype frequencies, including along the French Mediterranean coast where the present study takes place (Boissin et al. 2015; Cahill et al. 2017; Penant et al. 2013). Moreover, numerous cryptic species complexes, genetically distinct but morphologically indistinguishable, exhibit clear COI divergence (Cahill et al. 2024; Chenuil et al. 2019; Egea et al. 2016). Photographic analyses cannot resolve such variation, and most visually distinguishable taxa using our photographic protocol are widespread throughout the north‐western Mediterranean and therefore less likely to reflect geographic distances. Consequently, metabarcoding was expected to reflect geographic distances more clearly than photographic analyses, consistent with the stronger distance–decay relationships observed.

For environmental factors, expectations are less obvious. Because metabarcoding detects small, fast‐responding organisms, including unicellular taxa amplified by COI, community changes might be captured more rapidly by DNA than by imagery. This aligns with the classical view that microorganisms respond more strongly to environmental conditions than to geographic barriers, a concept captured by the phrase ‘everything is everywhere, but the environment selects’ (De Wit and Bouvier 2006; Van der Gast 2013). Under this paradigm, metabarcoding could be especially sensitive to environmental variation. However, photography better reflects dominant taxa that are likely well adapted to local conditions. They should, in principle, have abundances in photography that mirror biomass more faithfully than with metabarcoding. Read counts are imperfect indicators of abundance, owing to multiple sources of technical biases: (i) subsampling of the homogenized community may under‐represent rare taxa; (ii) DNA extraction efficiencies vary among taxa; and (iii) PCR amplification is taxon‐specific, with some lineages weakly or not amplified (Lamb et al. 2019; van der Loos and Nijland 2021). All else being equal, such biases should reduce the sensitivity of metabarcoding to environmental gradients. Yet our results contradict this expectation: metabarcoding exhibited stronger correlations with environmental variables than photography. This indicates that the far greater taxonomic richness recovered by metabarcoding compensates for its lower quantitative fidelity, enhancing its ability to detect environmentally driven changes in community composition (see also Deiner et al. 2017).

### 4.3 Implications and Perspectives for Routine Monitoring

Standardized ARMS protocols have become a cornerstone of global marine biodiversity observation, enabling comparable DNA‐ and photography‐based data across studies, regions and time (Ransome et al. 2017; Obst et al. 2020; Daraghmeh et al. 2025). However, substantial heterogeneity remains in sample treatment, molecular workflows and bioinformatic processing, potentially limiting interoperability across monitoring programmes and observatory networks (Jessop et al. 2026). Recent initiatives therefore emphasize standardized metadata, transparent workflows and FAIR practices to support data reuse and cross‐study integration (Klymus et al. 2024; Takahashi et al. 2025). These requirements are particularly critical for eDNA metabarcoding, because even minor changes in sampling, storage, sequencing or bioinformatic processing can cascade through analytical workflows and compromise inter‐study comparability (Zinger et al. 2019; van der Loos and Nijland 2021; Alberdi et al. 2018). Building on these challenges, our study refines key steps of ARMS‐based monitoring—from field deployment to molecular and photographic processing—to improve the consistency and long‐term comparability, providing the baseline on which more detailed sampling strategies can build.

#### 4.3.1 Sampling Design and Sampling Variance: Why Pooling All ARMS Faces Before Metabarcoding Should Be Avoided

A major difference between the four methods implemented in this study concerns sampling variance. Metabarcoding was more prone to stochasticity than photography, missing a larger fraction of taxa that are potentially detectable. In our dataset, this was evident from the markedly lower community similarity between adjacent sites and from the rarefaction curves further from their asymptotes compared with photographic data. Such variability is consistent with previous reports of eDNA detection heterogeneity (Zinger et al. 2019; Alberdi et al. 2018; Lamb et al. 2019). Because sequencing depth was not limiting (data not shown), this variance most likely reflects technical artefacts introduced during DNA extraction, PCR amplification and data filtering (Lamb et al. 2019; van der Loos and Nijland 2021; Zinger et al. 2019). By contrast, photographic analysis—although based on 64 randomly drawn points per image and thus theoretically subject to some sampling errors—missed far fewer taxa and yielded more consistent estimates of local similarity. These results strongly argue against pooling all sessile fractions from a single ARMS prior to metabarcoding. Indeed, the similarity of adjacent sites reached values as low as 5% in pooled faces metabarcoding, compared with 20% for separated‐faces metabarcoding and 50% for photographic analyses.

Analysing the 17 faces of a single ARMS per site separately increased the number of detected mOTUs by more than 2.4‐fold compared with pooling all sessile fractions from 22 ARMS (across 10 sites). The gain in diversity was even higher with the design separating five structural face categories across three ARMS per site. In addition, separating structural face categories may also reduce diversity loss caused by DNA degradation or PCR inhibition from bioactive compounds unevenly distributed among sessile taxa (Leray and Knowlton 2015). Shifts in phylum representation between pooled and separated designs further suggest that DNA extraction and amplification efficiencies vary with sample composition (see also Lamb et al. 2019; van der Loos and Nijland 2021). This hypothesis—namely that separating samples into structural face categories improves site characterization beyond the mere reduction of sampling variance—is also supported by the observation that community composition based on the five structural face categories captured geographic and environmental variation more strongly than the 17‐face design, even though it was based on fewer samples (137 vs. 166 across 10 sites). These results show that sampling design strongly influences both detected diversity and the identification of ecological drivers, making it a critical component of monitoring programmes. Our findings challenge the standard NOAA protocol, which recommends three ARMS units per site with pooled sessile fractions. Given the very low correlations we observed between community differentiation and geographic or environmental distances in pooled designs (Figure 8), it is unlikely that NOAA protocol (based on three pooled ARMS per site) would perform substantially better than the two pooled ARMS per site analysed here. Because separating 17 sessile samples per ARMS entails substantial extra work, and because the five‐category scheme provided an even clearer ecological signal, we subsequently adopted the intermediate solution for the SEAMoBB project: pooling faces into five structural categories based on orientation and compartmentalization, while retaining three ARMS per site. We considered including plate number (i.e., distance from the seafloor) as an additional structural factor. However, preliminary analyses showed no consistent pattern (data not shown), and plate number cannot be fully crossed—with respect to statistical design—with orientation and compartmentalization. Overall, this modified protocol offers a balanced compromise between logistical effort and ecological resolution.

#### 4.3.2 Refining Photographic Analysis: Greater Precision With Minimal Additional Effort

Photography‐based analyses also offer room for methodological refinement. Most ARMS image studies have relied on 50 random points per plate face, following a comparative analysis indicating that this density is generally sufficient. By increasing the density to 64 points, we improved precision without a meaningful increase in processing time, and found that photographic data were much less prone to sampling variance than metabarcoding, because adjacent sites harboured more similar communities (see also David et al. 2019). Although not formally quantified, increasing point density from 50 to 64 required little additional effort while improving abundance estimates. Nevertheless, further gains are likely to come from improving image quality and resolution, which would require higher‐grade cameras and greater digital‐storage capacity, rather than from further increasing point density. Expanding the number of taxonomic labels can enhance detection of changes in community structure, biomass and anthropogenic impacts (PERMANOVA tests, not shown), but it also increases training time and inter‐expert variability (see also Beijbom et al. 2015; Williams et al. 2019). Looking ahead, artificial intelligence–assisted image recognition is expected to accelerate annotation, improve consistency and reduce expert bias (Bravo et al. 2021; Raphael et al. 2020; Trotter et al. 2025).

#### 4.3.3 Traceable, Quality‐Controlled Bioinformatics

Long‐term eDNA monitoring requires datasets that remain comparable across sites, seasons and sequencing runs through rigorous quality control and standardization (Pawlowski et al. 2018; Petit‐Marty et al. 2023; Rimet et al. 2025). International standards and recent reviews emphasize bioinformatic workflows that minimize false positives and false negatives, ensure traceability, and support reproducible large‐scale comparisons (Pawlowski et al. 2018; Leese et al. 2018; Hakimzadeh et al. 2024; Rimet et al. 2025). The bioinformatic considerations raised by Turon et al. (2025) reinforce these requirements, showing that temporal trends can be confounded by technical discrepancies across sequencing runs or workflows and thus emphasizing the need for control‐driven, fully traceable pipelines.

In this context, the VTAM pipeline proved crucial as it explicitly integrates negative controls, mock communities and technical replicates to optimize filtering parameters, thereby reducing false positives and false negatives and ensuring full traceability at every filtering step (González et al. 2023). By adopting this validated pipeline, we ensured that the strong biogeographic and environmental patterns revealed by our face‐by‐face metabarcoding reflect true biological signals rather than analytical artefacts. This conclusion is further supported by recent studies demonstrating that VTAM‐filtered metabarcoding can generate highly validated data for fine‐scale, hypothesis‐driven analyses of community assembly and ecological interactions across aquatic ecosystems (e.g., Esposito et al. 2022; Ruiz et al. 2025; Villsen et al. 2025). VTAM provides a robust framework for ARMS metabarcoding by integrating negative controls, mock communities, technical replicates and transparent parameter optimization. This approach improves data comparability across sites and sequencing runs while aligning with recent FAIR data and reporting recommendations for environmental metabarcoding (Klymus et al. 2024; Takahashi et al. 2025), thereby supporting interoperability among large‐scale biodiversity observatories.

#### 4.3.4 Costs and Scalability

Cost and time considerations reinforce these conclusions. Assigning 64 points per image required only about 4–8 min for a trained analyst, so processing our 166 images amounted to less than 1 week of full‐time work after an initial 10‐day training period. By contrast, metabarcoding required roughly 1 month of benchtop labour for ~160 samples and about €6000 for reagents, consumables and sequencing, half of which was for high‐throughput sequencing. The adoption of Illumina NovaSeq sequencing has reduced per‐sample sequencing costs more than tenfold, but exploiting these economies of scale requires pooling sufficient libraries to fill sequencing runs, which may introduce delays that must be balanced against monitoring frequency. Similar trade‐offs between detection sensitivity and economic efficiency have been demonstrated in other eDNA studies, where laboratory costs can offset field savings and strongly influence optimal survey design (Smart et al. 2016). Broader reviews likewise stress that the choice of sampling and processing methods remains a major determinant of cost, throughput and inter‐study comparability in benthic metabarcoding (Pawlowski et al. 2022), underlining the need to balance detection sensitivity with cost efficiency when designing long‐term observatories. Overall, field effort and processing time are likely to remain the main constraints for large‐scale monitoring. Sustainable implementation will therefore depend on balancing ecological resolution with logistical effort and on shared infrastructures that ensure cost‐efficiency and data comparability across regions and monitoring programmes.

## 5 Conclusion

By coupling high‐resolution photographic validation with a control‐driven metabarcoding workflow, this study demonstrates that retaining ARMS microhabitat structure substantially improves biodiversity assessment. Analysing structural face categories increases sensitivity while maintaining manageable effort, providing a practical and scalable design for marine biodiversity observatories. These methodological refinements align with initiatives such as ARMS‐MBON and OBON and support standardized biodiversity monitoring across space and time.

## Author Contributions

A.C. and V.D. conceived and designed the study. A.C., A.H., D.G. and F.Z. chose the sampling sites. A.C., A.H., C.M., D.G., E.B., F.L., Fl.M., F.Z., L.V., L.P., M.S., P.M., S.C., S.R., T.L., V.C. and V.R. performed the fieldwork (scuba‐diving) and/or ARMS processing (fractioning, scraping, fixation and photography). V.D. conceived and developed the metabarcoding protocol. Fl.M., Fa.M. and C.C. did the molecular work. E.M. did the bioinformatics. J.M.G.O. provided environmental variables for all sites. A.C., E.B., D.G., M.L. and S.R. developed the protocol and/or contributed to the analyses of photography. L.P. trained the consortium members on ARMS processing. A.C. performed all statistical analyses and figures. A.C. and V.D. wrote the original draft. E.B., E.M., J.M.G.O., P.M., T.L., S.R. and V.R. contributed to further writing and editing. All authors approved the final version of the manuscript.

## Funding

This work was supported by the MONITOBEN and MONITOBEN‐2 projects, funded by EMBRC‐France (OOB_EMBRC FR_AAP2018_n°2179), and by the SEAMoBB project (Solutions for sEmi‐Automated Monitoring of Benthic Biodiversity), co‐funded by ERA‐Net Mar‐TERA (id. 145), the French National Research Agency (ANR; Grants ANR‐17‐MART0001‐01, ANR‐17‐MART0001‐02 and ANR‐17‐MART0001‐03), and the Centro para el Desarrollo Tecnológico e Industrial (CDTI, Spain; Project SERA‐20181031 ‘Soluciones para el Monitoreo Semi‐Automático de la Biodiversidad Bentónica’). V.R. acknowledges partial financial support from the European Space Agency (ESA Contract No. 4000141547/23/I‐DT) through the 4DMED‐Sea project. The project leading to this publication has received funding from European FEDER Fund under project 1166‐39417.

## Conflicts of Interest

The authors declare no conflicts of interest.

## Supporting information


**Figure S1:** Correlation plots of α‐ and β‐diversity among photographic and metabarcoding datasets at the 10 sites level. Pairwise correlations of α‐diversity (upper matrices) and β‐diversity (lower matrices) between datasets defined by three taxonomic‐rank levels for both photographic and metabarcoding data, drawn by the R package corrplot (version 0.95). Correlations on Spearman's rank coefficients were similar to Pearson's correlations. For α‐diversity, only matrix cells with correlation coefficients ≥ 0.69 are significant at *p* < 0.05, indicating that only metabarcoding data aggregated at the class level are positively correlated with photographic data for site richness. For β‐diversity, the threshold is 0.294. The metabarcoding dataset used here corresponds to the 17‐faces design (for relevant comparisons with photography).

**File S1:** File of several table sheets containing assemblage data, metadata, and taxonomy information for photography and metabarcoding.

**File S2:** Optimization of DNA extraction and PCR amplification protocols. Details of the protocols tested and compared to optimize metabarcoding, including DNA extraction, and PCR steps.

**File S3:** Parameters and the numbers of false positives and false negatives identified by the VTAM pipeline for each sequencing run.

**File S4:** Mantel and partial Mantel tests of community dissimilarity versus geographic distance and/or environmental variables for all assemblage datasets.

**File S5:** Mantel tests of community dissimilarity at different taxonomic resolution with geographic distance for all datasets.

**Table S1:** Study sites and sampling details for 2019. For each site, the table reports depth, geographical coordinates (decimal latitude and longitude), dates of immersion and emersion. In each site in 2019, three ARMS were analysed the same way: for the sessile fraction of each ARMS, faces were scraped and grouped according to their orientation and compartmentation into five categories (see the methods), then five DNA extractions and metabarcoding were carried out as explained in the methods. Two sites (BAN1 and VIL3) are reported here for information on the SEAMoBB project, but were not included in analyses presented in this study, to ensure comparability with photography and separate face metabarcoding.

## Data Availability

Sequence data have been deposited in the NCBI Sequence Read Archive (SRA) under the BioProject ID PRJNA1231031. Due to SRA submission requirements, archived FASTQ files correspond to demultiplexed and primer‐trimmed reads. The complete bioinformatic workflow used in this study, including read merging, demultiplexing, trimming, VTAM filtering steps and associated scripts for statistical analyses, is fully documented. Scripts and metadata files are available on GitHub (https://github.com/meglecz/seamobb_photo_metabarcoding), and large non‐demultiplexed FASTQ files and the custom reference database used for taxonomic assignment are archived in Zenodo (doi: https://doi.org/10.5281/zenodo.20344491). Occurrence data have been submitted to GBIF (https://doi.org/10.15468/s9dtvr).

## References

[men70188-bib-0001] Alberdi, A. , O. Aizpurua , M. T. P. Gilbert , and K. Bohmann . 2018. “Scrutinizing Key Steps for Reliable Metabarcoding of Environmental Samples.” Methods in Ecology and Evolution 9, no. 1: 134–147. 10.1111/2041-210X.12849.

[men70188-bib-0002] Anderson, M. J. , R. N. Gorley , and K. R. Clarke . 2008. PERMANOVA+ for PRIMER: Guide to Software and Statistical Methods, 218. PRIMER‐E Ltd.

[men70188-bib-0003] Andújar, C. , P. Arribas , D. W. Yu , A. P. Vogler , and B. C. Emerson . 2018. “Why the COI Barcode Should Be the Community DNA Metabarcoding Marker of Choice.” Molecular Ecology 27, no. 20: 3968–3975. 10.1111/mec.14844. 30129071

[men70188-bib-0004] Appeltans, W. , S. T. Ahyong , G. Anderson , et al. 2012. “The Magnitude of Global Marine Species Diversity.” Current Biology 22, no. 23: 2189–2202. 10.1016/j.cub.2012.09.036. 23159596

[men70188-bib-0005] Aylagas, E. , Á. Borja , X. Irigoien , and N. Rodríguez‐Ezpeleta . 2016. “Benchmarking DNA Metabarcoding for Biodiversity‐Based Monitoring and Assessment.” Frontiers in Marine Science 3: 96. 10.3389/fmars.2016.00096.

[men70188-bib-0006] Beijbom, O. , P. J. Edmunds , C. Roelfsema , et al. 2015. “Towards Automated Annotation of Benthic Survey Images: Variability of Human Experts and Operational Modes of Automation.” PLoS One 10, no. 7: e0130312. 10.1371/journal.pone.0130312. 26154157 PMC4496057

[men70188-bib-0007] Bianchi, C. N. , A. Azzola , S. Cocito , et al. 2022. “Biodiversity Monitoring in Mediterranean Marine Protected Areas: Scientific and Methodological Challenges.” Diversity 14, no. 1: 43. 10.3390/d14010043.

[men70188-bib-0008] Boissin, E. , E. Egea , J.‐P. Féral , and A. Chenuil . 2015. “Contrasting Population Genetic Structures in *Amphipholis squamata* , a Complex of Brooding, Self‐Reproducing Sister Species Sharing Life History Traits.” Marine Ecology Progress Series 539: 165–177. 10.3354/meps11480.

[men70188-bib-0009] Brandt, M. I. , F. Pradillon , B. Trouche , et al. 2021. “Evaluating Sediment and Water Sampling Methods for the Estimation of Deep‐Sea Biodiversity Using Environmental DNA.” Scientific Reports 11: 7856. 10.1038/s41598-021-86396-8. 33846371 PMC8041860

[men70188-bib-0010] Brandt, M. I. , B. Trouche , L. Quintric , et al. 2021. “Bioinformatic Pipelines Combining Denoising and Clustering Tools Allow for More Comprehensive Prokaryotic and Eukaryotic Metabarcoding.” Molecular Ecology Resources 21, no. 6: 1904–1921. 10.1111/1755-0998.13398. 33835712

[men70188-bib-0011] Bravo, G. , N. Moity , E. Londoño‐Cruz , et al. 2021. “Robots Versus Humans: Automated Annotation Accurately Quantifies Essential Ocean Variables of Rocky Intertidal Functional Groups and Habitat State.” Frontiers in Marine Science 8: 691313. 10.3389/fmars.2021.691313.

[men70188-bib-0012] Cahill, A. E. , A. De Jode , S. Dubois , et al. 2017. “A Multispecies Approach Reveals Hot Spots and Cold Spots of Diversity and Connectivity in Invertebrate Species With Contrasting Dispersal Modes.” Molecular Ecology 26, no. 23: 6563–6577. 10.1111/mec.14389. 29087018

[men70188-bib-0013] Cahill, A. E. , E. Meglécz , and A. Chenuil . 2024. “Scientific History, Biogeography, and Biological Traits Predict Presence of Cryptic or Overlooked Species.” Biological Reviews 99, no. 2: 546–561. 10.1111/brv.13034. 38049930

[men70188-bib-0014] Chao, A. , N. J. Gotelli , T. C. Hsieh , et al. 2014. “Rarefaction and Extrapolation With Hill Numbers: A Framework for Sampling and Estimation in Species Diversity Studies.” Ecological Monographs 84, no. 1: 45–67. 10.1890/13-0133.1.

[men70188-bib-0015] Chen, Q. , O. Beijbom , S. Chan , J. Bouwmeester , and D. Kriegman . 2021. “A New Deep Learning Engine for Coralnet.” In *Proceedings of the IEEE/CVF International Conference on Computer Vision*. 3693–3702.

[men70188-bib-0016] Chenuil, A. , A. E. Cahill , N. Délémontey , E. Du Salliant du Luc , and H. Fanton . 2019. “Problems and Questions Posed by Cryptic Species. A Framework to Guide Future Studies.” In From Assessing to Conserving Biodiversity: Conceptual and Practical Challenges, edited by E. Casetta , J. Marques da Silva , and D. Vecchi , 77–106. Springer. 10.1007/978-3-030-10991-2_4.

[men70188-bib-0017] Clarke, K. R. , and R. N. Gorley . 2015. PRIMER v7: User Manual/Tutorial. 3rd ed, 296. PRIMER‐E Ltd.

[men70188-bib-0018] Coll, M. , C. Piroddi , J. Steenbeek , et al. 2010. “The Biodiversity of the Mediterranean Sea: Estimates, Patterns, and Threats.” PLoS One 5, no. 8: e11842. 10.1371/journal.pone.0011842. 20689844 PMC2914016

[men70188-bib-0019] Collins, R. A. , J. Bakker , O. S. Wangensteen , et al. 2019. “Non‐Specific Amplification Compromises Environmental DNA Metabarcoding With COI.” Methods in Ecology and Evolution 10, no. 11: 1985–2001. 10.1111/2041-210X.13276.

[men70188-bib-0020] Corse, E. , E. Meglécz , G. Archambaud , et al. 2017. “A From‐Benchtop‐To‐Desktop Workflow for Validating HTS Data and for Taxonomic Identification in Diet Metabarcoding Studies.” Molecular Ecology Resources 17, no. 6: e146–e159. 10.1111/1755-0998.12703. 28776936

[men70188-bib-0089] Costello, M. J. , R. M. May , and N. E. Stork . 2013. “Can We Name Earth's Species Before they Go Extinct?” Science 339 no. 6118: 413–416. 10.1126/science.1230318 23349283

[men70188-bib-0022] Couëdel, M. , A. Dettai , M. M. Guillaume , et al. 2023. “New Insights Into the Diversity of Cryptobenthic *Cirripectes* Blennies in the Mascarene Archipelago Sampled Using Autonomous Reef Monitoring Structures (ARMS).” Ecology and Evolution 13, no. 3: e9850. 10.1002/ece3.9850. 36937067 PMC10019914

[men70188-bib-0023] Cowie, R. H. , P. Bouchet , and B. Fontaine . 2022. “The Sixth Mass Extinction: Fact, Fiction or Speculation?” Biological Reviews 97, no. 2: 640–663. 10.1111/brv.12816. 35014169 PMC9786292

[men70188-bib-0024] Danovaro, R. , J. Aguzzi , E. Fanelli , et al. 2017. “An Ecosystem‐Based Deep‐Ocean Strategy.” Science 355, no. 6324: 452–454. 10.1126/science.aah7178. 28154032

[men70188-bib-0025] Danovaro, R. , L. Carugati , M. Berzano , et al. 2016. “Implementing and Innovating Marine Monitoring Approaches for Assessing Marine Environmental Status.” Frontiers in Marine Science 3: 213. 10.3389/fmars.2016.00213.

[men70188-bib-0026] Daraghmeh, N. , K. Exter , J. Pagnier , et al. 2025. “A Long‐Term Ecological Research Data Set From the Marine Genetic Monitoring Program ARMS‐MBON 2018–2020.” Molecular Ecology Resources 25, no. 4: e14073. 10.1111/1755-0998.14073. 39887645 PMC11969632

[men70188-bib-0027] David, R. , M. C. Uyarra , S. Carvalho , et al. 2019. “Lessons From Photo Analyses of Autonomous Reef Monitoring Structures as Tools to Detect (Bio‐)geographical, Spatial, and Environmental Effects.” Marine Pollution Bulletin 141: 420–429. 10.1016/j.marpolbul.2019.02.066. 30955752

[men70188-bib-0028] De Wit, R. , and T. Bouvier . 2006. “‘Everything Is Everywhere, but, the Environment Selects’; What Did Baas Becking and Beijerinck Really Say?” Environmental Microbiology 8, no. 4: 755–758. 10.1111/j.1462-2920.2006.01017.x. 16584487

[men70188-bib-0029] Deiner, K. , H. M. Bik , E. Mächler , et al. 2017. “Environmental DNA Metabarcoding: Transforming How We Survey Animal and Plant Communities.” Molecular Ecology 26, no. 21: 5872–5895. 10.1111/mec.14350. 28921802

[men70188-bib-0030] Díaz, S. , J. Settele , E. S. Brondízio , et al. 2019a. “Pervasive Human‐Driven Decline of Life on Earth Points to the Need for Transformative Change.” Science 366, no. 6471: eaax3100. 10.1126/science.aax3100. 31831642

[men70188-bib-0031] Díaz, S. M. , J. Settele , E. Brondízio , et al. 2019b. The Global Assessment Report on Biodiversity and Ecosystem Services: Summary for Policy Makers, 56. IPBES.

[men70188-bib-0032] Duarte, C. M. , S. Agusti , E. Barbier , et al. 2020. “Rebuilding Marine Life.” Nature 580, no. 7801: 39–51. 10.1038/s41586-020-2146-7. 32238939

[men70188-bib-0033] Egea, E. , B. David , T. Chone , B. Laurin , J. P. Feral , and A. Chenuil . 2016. “Morphological and Genetic Analyses Reveal a Cryptic Species Complex in the Echinoid *Echinocardium cordatum* and Rule Out a Stabilizing Selection Explanation.” Molecular Phylogenetics and Evolution 94: 207–220. 10.1016/j.ympev.2015.07.023. 26265259

[men70188-bib-0034] Escudier, R. , E. Clementi , M. Omar , et al. 2020. Mediterranean Sea Physical Reanalysis (CMEMS MED‐Currents) (Version 1) [Data Set]. Copernicus Monitoring Environment Marine Service (CMEMS). 10.25423/CMCC/MEDSEA_MULTIYEAR_PHY_006_004_E3R1.

[men70188-bib-0035] Esposito, A. , P. Sasal , É. Clua , E. Meglécz , and C. Clerissi . 2022. “Shark Provisioning Influences the Gut Microbiota of the Black‐Tip Reef Shark in French Polynesia.” Fishes 7, no. 6: 312. 10.3390/fishes7060312.

[men70188-bib-0036] Estes, M., Jr. , C. Anderson , W. Appeltans , et al. 2021. “Enhanced Monitoring of Life in the Sea Is a Critical Component of Conservation Management and Sustainable Economic Growth.” Marine Policy 132: 104699. 10.1016/j.marpol.2021.104699.

[men70188-bib-0037] González, A. , V. Dubut , E. Corse , et al. 2023. “VTAM: A Robust Pipeline for Validating Metabarcoding Data Using Controls.” Computational and Structural Biotechnology Journal 21: 1151–1156. 10.1016/j.csbj.2023.01.034. 36789260 PMC9918390

[men70188-bib-0038] GRASS Development Team , M. Landa , M. Neteler , et al. 2025. “GRASS GIS (8.4.1).” *Zenodo*. 10.5281/zenodo.14918754.

[men70188-bib-0039] Hajibabaei, M. , T. M. Porter , C. V. Robinson , D. J. Baird , S. Shokralla , and M. T. Wright . 2019. “Watered‐Down Biodiversity? A Comparison of Metabarcoding Results From DNA Extracted From Matched Water and Bulk Tissue Biomonitoring Samples.” PLoS One 14, no. 12: e0225409. 10.1371/journal.pone.0225409. 31830042 PMC6907778

[men70188-bib-0040] Hakimzadeh, A. , A. Abdala Asbun , D. Albanese , et al. 2024. “A Pile of Pipelines: An Overview of the Bioinformatics Software for Metabarcoding Data Analyses.” Molecular Ecology Resources 24, no. 5: e13847. 10.1111/1755-0998.13847. 37548515 PMC10847385

[men70188-bib-0041] Halpern, B. S. , M. Frazier , J. Potapenko , et al. 2015. “Spatial and Temporal Changes in Cumulative Human Impacts on the World's Ocean.” Nature Communications 6: 7615. 10.1038/ncomms8615. PMC4510691 26172980

[men70188-bib-0042] Hintikka, S. , J. E. Carlsson , and J. Carlsson . 2022. “The Bacterial Hitchhiker's Guide to COI: Universal Primer‐Based COI Capture Probes Fail to Exclude Bacterial DNA, but 16S Capture Leaves Metazoa Behind.” Metabarcoding and Metagenomics 6: e80416. 10.3897/mbmg.6.80416.

[men70188-bib-0043] Hsieh, T. C. , K. H. Ma , and A. Chao . 2016. “iNEXT: An R Package for Rarefaction and Extrapolation of Species Diversity (Hill Numbers).” Methods in Ecology and Evolution 7, no. 12: 1451–1456. 10.1111/2041-210X.12613.

[men70188-bib-0044] Jessop, A. , M. Steyaert , N. Daraghmeh , et al. 2026. “A Review of Autonomous Reef Monitoring Structures (ARMS) for Monitoring Hard‐Bottom Benthic Biodiversity.” Methods in Ecology and Evolution 17, no. 2: 435–455. 10.1111/2041-210x.70194.

[men70188-bib-0045] Klymus, K. E. , J. D. Baker , C. L. Abbott , et al. 2024. “The MIEM Guidelines: Minimum Information for Reporting of Environmental Metabarcoding Data.” Metabarcoding and Metagenomics 8: e128689. 10.3897/mbmg.8.128689.

[men70188-bib-0046] Laikre, L. , S. Hoban , M. W. Bruford , et al. 2020. “Post‐2020 Goals Overlook Genetic Diversity.” Science 367, no. 6482: 1083–1085. 10.1126/science.abb2748 32139534

[men70188-bib-0047] Lamb, P. D. , E. Hunter , J. K. Pinnegar , S. Creer , R. G. Davies , and M. I. Taylor . 2019. “How Quantitative Is Metabarcoding: A Meta‐Analytical Approach.” Molecular Ecology 28, no. 2: 420–430. 10.1111/mec.14920. 30408260 PMC7379500

[men70188-bib-0049] Leese, F. , F. Altermatt , A. Bouchez , et al. 2018. “Why We Need Sustainable Networks Bridging Countries, Disciplines, Cultures and Generations for Aquatic Biomonitoring 2.0: A Perspective Derived From the DNAqua‐Net COST Action.” In Next Generation Biomonitoring: Part 1, edited by D. A. Bohan , A. J. Dumbrell , G. Woodward , and M. Jackson , 63–99. Academic Press.

[men70188-bib-0050] Legrand, T. , A. Chenuil , E. Ser‐Giacomi , S. Arnaud‐Haond , N. Bierne , and V. Rossi . 2022. “Spatial Coalescent Connectivity Through Multi‐Generation Dispersal Modelling Predicts Gene Flow Across Marine Phyla.” Nature Communications 13: 5861. 10.1038/s41467-022-33499-z. PMC9532449 36195609

[men70188-bib-0051] Leray, M. , and N. Knowlton . 2015. “DNA Barcoding and Metabarcoding of Standardized Samples Reveal Patterns of Marine Benthic Diversity.” Proceedings of the National Academy of Sciences of the United States of America 112, no. 7: 2076–2081. 10.1073/pnas.1424997112. 25646458 PMC4343139

[men70188-bib-0052] Mahé, F. , L. Czech , A. Stamatakis , et al. 2022. “Swarm v3: Towards Tera‐Scale Amplicon Clustering.” Bioinformatics 38, no. 1: 267–269. 10.1093/bioinformatics/btab493. PMC8696092 34244702

[men70188-bib-0053] Meglécz, E. 2023a. “COInr and mkCOInr: Building and Customizing a Nonredundant Barcoding Reference Database From BOLD and NCBI Using a Semi‐Automated Pipeline.” Molecular Ecology Resources 23, no. 4: 933–945. 10.1111/1755-0998.13756. 36656075

[men70188-bib-0054] Meglécz, E. 2023b. “mkLTG: A Command‐Line Tool for Taxonomic Assignment of Metabarcoding Sequences Using Variable Identity Thresholds.” Biologia Futura 74: 369–375. 10.1007/s42977-024-00201-x. 38300415

[men70188-bib-0055] Miloslavich, P. , N. J. Bax , S. E. Simmons , et al. 2018. “Essential Ocean Variables for Global Sustained Observations of Biodiversity and Ecosystem Changes.” Global Change Biology 24, no. 6: 2416–2433. 10.1111/gcb.14108. 29623683

[men70188-bib-0057] Obst, M. , K. Exter , A. L. Allcock , et al. 2020. “A Marine Biodiversity Observation Network for Genetic Monitoring of Hard‐Bottom Communities (ARMS‐MBON).” Frontiers in Marine Science 7: 572680. 10.3389/fmars.2020.572680.

[men70188-bib-0058] Obura, D. O. 2012. “The Diversity and Biogeography of Western Indian Ocean Reef‐Building Corals.” PLoS One 7, no. 9: e45013. 10.1371/journal.pone.0045013. 23028737 PMC3446983

[men70188-bib-0059] Pagnier, J. , N. Daraghmeh , and M. Obst . 2025. “Using the Long‐Term Genetic Monitoring Network ARMS‐MBON to Detect Marine Non‐Indigenous Species Along the European Coasts.” Biological Invasions 27: 77. 10.1007/s10530-024-03503-2.

[men70188-bib-0060] Paradis, E. , S. Blomberg , B. Bolker , et al. 2019. “ape: Analyses of Phylogenetics and Evolution.” R Package Version 2. https://cran.r‐project.org/package=ape.

[men70188-bib-0061] Pawlowski, J. , K. Bruce , K. Panksep , et al. 2022. “Environmental DNA Metabarcoding for Benthic Monitoring: A Review of Sediment Sampling and DNA Extraction Methods.” Science of the Total Environment 818: 151783. 10.1016/j.scitotenv.2021.151783. 34801504

[men70188-bib-0062] Pawlowski, J. , M. Kelly‐Quinn , F. Altermatt , et al. 2018. “The Future of Biotic Indices in the Ecogenomic Era: Integrating (e)DNA Metabarcoding in Biological Assessment of Aquatic Ecosystems.” Science of the Total Environment 637–638: 1295–1310. 10.1016/j.scitotenv.2018.05.002. 29801222

[men70188-bib-0063] Pearman, J. K. , G. Chust , E. Aylagas , et al. 2020. “Pan‐Regional Marine Benthic Cryptobiome Biodiversity Patterns Revealed by Metabarcoding Autonomous Reef Monitoring Structures.” Molecular Ecology 29, no. 24: 4882–4897. 10.1111/mec.15692. 33063375

[men70188-bib-0064] Pearman, J. K. , M. Leray , R. Villalobos , et al. 2018. “Cross‐Shelf Investigation of Coral Reef Cryptic Benthic Organisms Reveals Diversity Patterns of the Hidden Majority.” Scientific Reports 8: 8090. 10.1038/s41598-018-26332-5. 29795402 PMC5967342

[men70188-bib-0065] Penant, G. , D. Aurelle , J.‐P. Feral , and A. Chenuil . 2013. “Planktonic Larvae Do Not Ensure Gene Flow in the Edible Sea Urchin *Paracentrotus lividus* .” Marine Ecology Progress Series 480: 155–170. 10.3354/meps10194.

[men70188-bib-0066] Petit‐Marty, N. , L. Casas , and F. Saborido‐Rey . 2023. “State‐of‐the‐Art of Data Analyses in Environmental DNA Approaches Towards Its Applicability to Sustainable Fisheries Management.” Frontiers in Marine Science 10: 1061530. 10.3389/fmars.2023.1061530.

[men70188-bib-0067] Plaisance, L. , M. J. Caley , R. E. Brainard , and N. Knowlton . 2011. “The Diversity of Coral Reefs: What Are We Missing?” PLoS One 6, no. 10: e25026. 10.1371/journal.pone.0025026. 22022371 PMC3192706

[men70188-bib-0068] R Core Development Team . 2023. R: A Language and Environment for Statistical Computing. R Foundation for Statistical Computing. http://www.r‐project.org.

[men70188-bib-0069] Ransome, E. , J. B. Geller , M. A. Timmers , et al. 2017. “The Importance of Standardization for Biodiversity Comparisons: A Case Study Using Autonomous Reef Monitoring Structures (ARMS) and Metabarcoding to Measure Cryptic Diversity on Mo'orea Coral Reefs, French Polynesia.” PLoS One 12, no. 4: e0175066. 10.1371/journal.pone.0175066. 28430780 PMC5400227

[men70188-bib-0070] Raphael, A. , Z. Dubinsky , D. Iluz , and N. S. Netanyahu . 2020. “Neural Network Recognition of Marine Benthos and Corals.” Diversity 12, no. 1: 29. 10.3390/d12010029. PMC7395127 32737327

[men70188-bib-0071] Rimet, F. , C. Lemonnier , B. Alric , et al. 2025. “Omics to Study and Manage Aquatic Environments: A Snapshot From the AquaEcOmics Meeting.” Molecular Ecology 34, no. 17: e70041. 10.1111/mec.70041. 40698877

[men70188-bib-0072] Ruiz, C. , J. Vacelet , F. Corallo , et al. 2025. “Cellular and Molecular Microbial Diversity of the Mediterranean Sponge *Agelas oroides* (Demospongiae, Agelasida).” Marine Biology 172: 69. 10.1007/s00227-025-04627-2.

[men70188-bib-0073] Smart, A. S. , A. R. Weeks , A. R. Van Rooyen , A. Moore , M. A. McCarthy , and R. Tingley . 2016. “Assessing the Cost‐Efficiency of Environmental DNA Sampling.” Methods in Ecology and Evolution 7, no. 11: 1291–1298. 10.1111/2041-210X.12598.

[men70188-bib-0074] Taberlet, P. , A. Bonin , L. Zinger , and É. Coissac . 2018. Environmental DNA: For Biodiversity Research and Monitoring, 253. Oxford University Press.

[men70188-bib-0075] Takahashi, M. , T. G. Frøslev , J. Paupério , et al. 2025. “A Metadata Checklist and Data Formatting Guidelines to Make eDNA FAIR (Findable, Accessible, Interoperable, and Reusable).” Environmental DNA 7, no. 3: e70100. 10.1002/edn3.70100.

[men70188-bib-0076] Teruzzi, A. , V. Di Biagio , L. Feudale , et al. 2021. Mediterranean Sea Biogeochemical Reanalysis (CMEMS MED‐Biogeochemistry, MedBFM3 System) (Version 1) [Data Set]. Copernicus Monitoring Environment Marine Service (CMEMS). 10.25423/CMCC/MEDSEA_MULTIYEAR_BGC_006_008_MEDBFM3.

[men70188-bib-0077] Thomasdotter, A. , P. Shum , F. Mugnai , et al. 2023. “Spineless and Overlooked: DNA Metabarcoding of Autonomous Reef Monitoring Structures Reveals Intra‐ and Interspecific Genetic Diversity in Mediterranean Invertebrates.” Molecular Ecology Resources 23, no. 7: 1689–1705. 10.1111/1755-0998.13836. 37452608

[men70188-bib-0078] Trotter, C. , H. J. Griffiths , and R. J. Whittle . 2025. “Surveying the Deep: A Review of Computer Vision in the Benthos.” Ecological Informatics 86: 102989. 10.1016/j.ecoinf.2024.102989.

[men70188-bib-0079] Turon, X. , A. Antich , C. Palacin , K. Praebel , and O. S. Wangensteen . 2020. “From Metabarcoding to Metaphylogeography: Separating the Wheat From the Chaff.” Ecological Applications 30, no. 2: e02036. 10.1002/eap.2036. 31709684 PMC7078904

[men70188-bib-0080] Turon, X. , O. S. Wangensteen , J. Zarcero , C. Palacin , and A. Antich . 2025. “Advancing Marine Conservation: Metabarcoding and Metaphylogeography for a Multi‐Year Biomonitoring of Benthic Communities of National Parks Across Two Seas.” Diversity and Distributions 31, no. 11: e70121. 10.1111/ddi.70121.

[men70188-bib-0081] Van der Gast, C. J. 2013. “Microbial Biogeography and What Baas Becking Should Have Said.” Microbiology Today 40: 108–111.

[men70188-bib-0082] van der Loos, L. M. , and R. Nijland . 2021. “Biases in Bulk: DNA Metabarcoding of Marine Communities and the Methodology Involved.” Molecular Ecology 30, no. 13: 3270–3288. 10.1111/mec.15592. 32779312 PMC8359149

[men70188-bib-0083] Villsen, K. , G. Archambaud‐Suard , E. Meglécz , et al. 2025. “Disentangling the Effects of Biotic and Abiotic Dimensions of Ecological Opportunity on Individual Trophic Trait Variation.” Molecular Ecology 34, no. 20: e70115. 10.1111/mec.70115. 40988491 PMC12530292

[men70188-bib-0084] Wei, T. , V. Simko , M. Levy , Y. Xie , Y. Jin , and J. Zemla . 2017. “corrplot: Visualization of a Correlation Matrix.” R Package Version 0.84. https://cran.r‐project.org/package=corrplot.

[men70188-bib-0085] Wickham, H. , W. Chang , and M. H. Wickham . 2016. “ggplot2: Elegant Graphics for Data Analysis.” R Package Version 2. https://cran.r‐project.org/package=ggplot2.

[men70188-bib-0086] Williams, I. D. , C. S. Couch , O. Beijbom , et al. 2019. “Leveraging Automated Image Analysis Tools to Transform Our Capacity to Assess Status and Trends of Coral Reefs.” Frontiers in Marine Science 6: 222. 10.3389/fmars.2019.00222.

[men70188-bib-0087] Zinger, L. , A. Bonin , I. G. Alsos , et al. 2019. “DNA Metabarcoding—Need for Robust Experimental Designs to Draw Sound Ecological Conclusions.” Molecular Ecology 28, no. 8: 1857–1862. 10.1111/mec.15060. 31033079

