# Supplementary material for: Separating Faces in ARMS Metabarcoding Improves Marine Biodiversity Monitoring: A Comparison Across Protocols, Experimental Designs and Photographic Surveys
Source: Mol Ecol Resour. 2026 Sep 11;26(7):e70188. doi: 10.1111/1755-0998.70188 (PMC13564238; doi:10.1111/1755-0998.70188)
Supplement: Supplementary file 1 [file MEN-26-e70188-s003.pdf]

Results of Mantel tests from pairwise sites community dissimilarity compared with geographic distances (see methods) from metabarcoding and imaging data at varying levels of taxonomic aggregation

For the 17 separate faces datasets (one ARMS per site, year 2018):

dmotu :	distances among sites based on all mOTUs
dlab0 :	distances among sites based on all labels (38 labels)
dclass :	distances among sites after collapsing at class rank (w/o mOTUs unidentified at this rank)
dphylum :	distances among sites after collapsing at phylum rank (w/o mOTUs unidentified at this rank)
dlab2 :	distances among sites based on labelset2 (34 labels)
dphophyl :	distances among sites based on phylum labels (labels)

For other metabarcoding designs (distances among sites based on all mOTUs):

dmix :	pooled sessile fractions (2 ARMS per site, year 2018)
dmotu5t2019 :	five structural face categories (3 ARMS per site, year 2019)

```
> mantel(dmotu, ddgeo,permutations=99999)
Mantel statistic based on Pearson's product-moment correlation

Call:
mantel(xdis = dmotu, ydis = ddgeo, permutations = 99999)

Mantel statistic r: 0.7102
      Significance: 0.00024

Upper quantiles of permutations (null model):
  90%   95% 97.5%   99%
0.195 0.271 0.343 0.447
Permutation: free
Number of permutations: 99999

> mantel(dmotu, log(ddgeo+1),permutations=99999)
Mantel statistic based on Pearson's product-moment correlation

Call:
mantel(xdis = dmotu, ydis = log(ddgeo + 1), permutations = 99999)

Mantel statistic r: 0.7008
      Significance: 1e-05

Upper quantiles of permutations (null model):
  90%   95% 97.5%   99%
0.199 0.275 0.347 0.469
Permutation: free
Number of permutations: 99999

> mantel(dlab0, ddgeo,permutations=99999)
Mantel statistic based on Pearson's product-moment correlation

Call:
mantel(xdis = dlab0, ydis = ddgeo, permutations = 99999)

Mantel statistic r: 0.5008
      Significance: 0.00307

Upper quantiles of permutations (null model):
```

```
  90%   95% 97.5%   99% 
0.200 0.276 0.337 0.416 
Permutation: free
Number of permutations: 99999
```

> mantel(dlab0, log(ddgeo+1),permutations=99999)

```
Mantel statistic based on Pearson's product-moment correlation 

Call:
mantel(xdis = dlab0, ydis = log(ddgeo + 1), permutations = 99999) 

Mantel statistic r: 0.5496 
      Significance: 0.00045 

Upper quantiles of permutations (null model):
  90%   95% 97.5%   99% 
0.189 0.255 0.320 0.396 
Permutation: free
Number of permutations: 99999
```

> mantel(dmix, ddgeo,permutations=99999)

```
Mantel statistic based on Pearson's product-moment correlation 

Call:
mantel(xdis = dmix, ydis = ddgeo, permutations = 99999) 

Mantel statistic r: 0.2266 
      Significance: 0.06838 

Upper quantiles of permutations (null model):
  90%   95% 97.5%   99% 
0.202 0.249 0.288 0.330 
Permutation: free
Number of permutations: 99999
```

> mantel(dmix, log(ddgeo+1),permutations=99999) # r=0.1812, p=0.1359 NS encore pire pour p-value

```
Mantel statistic based on Pearson's product-moment correlation 

Call:
mantel(xdis = dmix, ydis = log(ddgeo + 1), permutations = 99999) 

Mantel statistic r: 0.1812 
      Significance: 0.13401 

Upper quantiles of permutations (null model):
  90%   95% 97.5%   99% 
0.216 0.286 0.340 0.393 
Permutation: free
Number of permutations: 99999
```

> mantel(dmotu5t2019, ddgeo,permutations=99999) # r=0.73, p=0.00004 BEST approche 5 types de faces (mais R proche de 17 faces) !

```
Mantel statistic based on Pearson's product-moment correlation 

Call:
mantel(xdis = dmotu5t2019, ydis = ddgeo, permutations = 99999) 

Mantel statistic r: 0.7335 
      Significance: 5e-05 
```

```
Upper quantiles of permutations (null model):
  90%   95% 97.5%   99%
0.189 0.259 0.350 0.476
Permutation: free
Number of permutations: 99999

> mantel(dmotu5t2019, log(ddgeo+1),permutations=99999)
Mantel statistic based on Pearson's product-moment correlation

Call:
mantel(xdis = dmotu5t2019, ydis = log(ddgeo + 1), permutations = 99999)

Mantel statistic r: 0.8366
      Significance: 2e-05

Upper quantiles of permutations (null model):
  90%   95% 97.5%   99%
0.192 0.267 0.369 0.493
Permutation: free
Number of permutations: 99999

> mantel(dclass, ddgeo,permutations=99999)
Mantel statistic based on Pearson's product-moment correlation

Call:
mantel(xdis = dclass, ydis = ddgeo, permutations = 99999)

Mantel statistic r: 0.542
      Significance: 0.00135

Upper quantiles of permutations (null model):
  90%   95% 97.5%   99%
0.202 0.273 0.326 0.417
Permutation: free
Number of permutations: 99999

> mantel(dclass, log(ddgeo+1),permutations=99999)
Mantel statistic based on Pearson's product-moment correlation

Call:
mantel(xdis = dclass, ydis = log(ddgeo + 1), permutations = 99999)

Mantel statistic r: 0.4337
      Significance: 0.00173

Upper quantiles of permutations (null model):
  90%   95% 97.5%   99%
0.163 0.217 0.273 0.338
Permutation: free
Number of permutations: 99999

> mantel(dphylum, ddgeo,permutations=99999)
Mantel statistic based on Pearson's product-moment correlation

Call:
mantel(xdis = dphylum, ydis = ddgeo, permutations = 99999)

Mantel statistic r: 0.3929
      Significance: 0.00947

Upper quantiles of permutations (null model):
  90%   95% 97.5%   99%
0.205 0.267 0.320 0.390
```

Permutation: free
Number of permutations: 99999

> mantel(dphylum, log(ddgeo+1),permutations=99999)
Mantel statistic based on Pearson's product-moment correlation

Call:
mantel(xdis = dphylum, ydis = log(ddgeo + 1), permutations = 99999)

Mantel statistic r: 0.3374
Significance: 0.004

Upper quantiles of permutations (null model):

90%	95%	97.5%	99%
0.153	0.204	0.249	0.296

Permutation: free
Number of permutations: 99999

> mantel(dlab2, ddgeo,permutations=99999)
Mantel statistic based on Pearson's product-moment correlation

Call:
mantel(xdis = dlab2, ydis = ddgeo, permutations = 99999)

Mantel statistic r: 0.4827
Significance: 0.00638

Upper quantiles of permutations (null model):

90%	95%	97.5%	99%
0.202	0.271	0.334	0.420

Permutation: free
Number of permutations: 99999

> mantel(dlab2, log(ddgeo+1),permutations=99999)
Mantel statistic based on Pearson's product-moment correlation

Call:
mantel(xdis = dlab2, ydis = log(ddgeo + 1), permutations = 99999)

Mantel statistic r: 0.4704
Significance: 0.00137

Upper quantiles of permutations (null model):

90%	95%	97.5%	99%
0.178	0.241	0.300	0.370

Permutation: free
Number of permutations: 99999

> mantel(dphophyl, ddgeo,permutations=99999)
Mantel statistic based on Pearson's product-moment correlation

Call:
mantel(xdis = dphophyl, ydis = ddgeo, permutations = 99999)

Mantel statistic r: 0.2369
Significance: 0.0744

Upper quantiles of permutations (null model):

90%	95%	97.5%	99%
0.211	0.270	0.318	0.368

Permutation: free
Number of permutations: 99999

```
> mantel(dphophyl, log(ddgeo+1),permutations=99999)
Mantel statistic based on Pearson's product-moment correlation

Call:
mantel(xdis = dphophyl, ydis = log(ddgeo + 1), permutations = 99999)

Mantel statistic r: 0.2669
      Significance: 0.01621

Upper quantiles of permutations (null model):
  90%   95% 97.5%   99%
0.157 0.206 0.243 0.290
Permutation: free
Number of permutations: 99999
```
