# Supplementary material for: Separating Faces in ARMS Metabarcoding Improves Marine Biodiversity Monitoring: A Comparison Across Protocols, Experimental Designs and Photographic Surveys
Source: Mol Ecol Resour. 2026 Sep 11;26(7):e70188. doi: 10.1111/1755-0998.70188 (PMC13564238; doi:10.1111/1755-0998.70188)
Supplement: Supplementary file 2 [file MEN-26-e70188-s001.docx]

Table S1: **Study sites and sampling details for 2019**

For each site, the table reports depth, geographical coordinates (decimal latitude and longitude), dates of immersion and emersion. In each site in 2019, three ARMS were analyzed the same way: for the sessile fraction of each ARMS, faces were scraped and grouped according to their orientation and compartmentation into five categories (see the methods), then five DNA extractions and metabarcoding were carried out as explained in the methods. Two sites (BAN1 and VIL3) are reported here for information on the SEAMoBB project, but were not included in analyses presented in this study, to ensure comparability with photography and separate face metabarcoding.

| **Site code** | **Site name** | **Region** | **Latitude** | **Longitude** | **Depth** | **Date in** | **Date out** |
| --- | --- | --- | --- | --- | --- | --- | --- |
| *BAN1* | *Port-vendres S* | *Banyuls-sur-Mer* | *42,51327* | *3,13743* | 17 m | *17/04/2018* | *20/02/2019* |
| BAN2 | Port-vendres N | Banyuls-sur-Mer | 42,52518 | 3,11050 | 17 m | 12/04/2018 | 19/02/2019 |
| BAN3 | Cerbère N | Banyuls-sur-Mer | 42,44818 | 3,17212 | 17 m | 19/03/2018 | 20/02/2019 |
| BAN4 | Cerbère S | Banyuls-sur-Mer | 42,44262 | 3,17405 | 17 m | 19/03/2018 | 20/02/2019 |
| MAR1 | Veyron | Marseille | 43,20683 | 5,25350 | 17,5 m | 07/05/2018 | 19/04/2019 |
| MAR2 | Digue des catalans | Marseille | 43,29167 | 5,34558 | 19 m | 05/03/2018 | 26/03/2019 |
| MAR3 | Niolon | Marseille | 43,34150 | 5,26525 | 16,5 m | 09/05/2018 | 26/03/2019 |
| MAR4 | Plane | Marseille | 43,19067 | 5,38233 | 17,5 m | 05/03/2018 | 19/04/2019 |
| VIL1 | Pointe de la gavinette | Villefranche-sur-Mer | 43,68683 | 7,32138 | 20 m | 13/03/2018 | 04/03/2019 |
| VIL2 | Phare du cap ferrat | Villefranche-sur-Mer | 43,67427 | 7,32803 | 18 m | 15/03/2018 | 06/03/2019 |
| *VIL3* | *Grotte à corail* | *Villefranche-sur-Mer* | *43,68945* | *7,30883* | 17 m | *13/03/2018* | *05/03/2019* |
| VIL4 | Cap ferrat est | Villefranche-sur-Mer | 43,67675 | 7,33508 | 18 m | 14/03/2018 | 06/03/2019 |
