# Supplementary material for: Separating Faces in ARMS Metabarcoding Improves Marine Biodiversity Monitoring: A Comparison Across Protocols, Experimental Designs and Photographic Surveys
Source: Mol Ecol Resour. 2026 Sep 11;26(7):e70188. doi: 10.1111/1755-0998.70188 (PMC13564238; doi:10.1111/1755-0998.70188)
Supplement: Supplementary file 3 [file MEN-26-e70188-s005.docx]

Figure S1 (to be continued next page) : **Correlation plots of α- and β-diversity among photographic and metabarcoding datasets at the 10 sites level.**

Pairwise correlations of α-diversity (upper matrices) and β-diversity (lower matrices) between datasets defined by three taxonomic-rank levels for both photographic and metabarcoding data, drawn by the R package corrplot (version 0.95). Correlations on Spearman’s rank coefficients were similar to Pearson’s correlations. For α-diversity, only matrix cells with correlation coefficients ≥ 0.69 are significant at *p* < 0.05, indicating that only metabarcoding data aggregated at the class level are positively correlated with photographic data for site richness. For β-diversity, the threshold is 0.294. The metabarcoding dataset used here corresponds to the 17-faces design (for relevant comparisons with photography).

Figure S1 (… end)
