# Supplementary material for: Separating Faces in ARMS Metabarcoding Improves Marine Biodiversity Monitoring: A Comparison Across Protocols, Experimental Designs and Photographic Surveys
Source: Mol Ecol Resour. 2026 Sep 11;26(7):e70188. doi: 10.1111/1755-0998.70188 (PMC13564238; doi:10.1111/1755-0998.70188)
Supplement: Supplementary file 4 [file MEN-26-e70188-s007.docx]

**Supplementary File S2** – **Optimization of DNA extraction and PCR amplification protocols**

**1. Overview**

Prior to the publication of the standardized workflow by Thomasdotter et al. (2023), optimization trials were conducted within the SEAMOBB project to evaluate the influence of DNA extraction and PCR amplification parameters on sequencing yield, taxonomic breadth, and reproducibility. Two DNA extraction kits and two polymerases were compared across representative sessile and mobile ARMS fractions to identify the most efficient and reproducible combination.

**2. Sampling and preparation of ARMS material**

Autonomous Reef Monitoring Structures (ARMS) consist of nine stacked PVC plates alternately separated by PVC corners (“open plates”) or cross-shaped spacers (“closed plates”), creating 17 exposed faces per unit. Sessile fractions were scraped, preserved in 96% ethanol, homogenized using a Moulinex LM300141 blender, and sieved through a 40 µm mesh. After each blending, both the blender jar and mesh were decontaminated for 20 min in bleach and rinsed with distilled water. Approximately 10 mL of dry filtrate were transferred into 50 mL Falcon tubes and filled with 96% ethanol.

Mobile fractions were obtained by filtering the water retained between plates through 2 mm, 500 µm, and 100 µm meshes. The 500 µm and 100 µm filtrates were retained for metabarcoding, homogenized by shaking, and subsampled twice (2 × 750 µL in 2 mL tubes). Samples were centrifuged (4 min, 12,000 × g), ethanol removed, and pellets dried in a Savant® DNA SpeedVac® DNA 120 (Thermo Fisher Scientific, MA, USA) at 45 °C for 45 min.

**3. DNA extraction trials**

Each of the six homogenized ARMS samples (four sessile, two mobile) was extracted at least twice, using two alternative DNA extraction kits: NucleoSpin® Soil Kit (Macherey-Nagel, Germany), and DNeasy *mericon*® Food Kit (Qiagen, Germany) (Figure A, Table A). Four samples were extracted twice with each kit, one of them four times with DNeasy *mericon*® Food Kit, yielding 24 DNA extracts. For the NucleoSpin® Soil Kit, the manufacturer’s protocol was followed using SL1 + SX lysis buffers. Mechanical disruption was performed using a TissueLyser II (Qiagen, Germany) (30 Hz, 5 min). After addition of buffer SL3, lysates were transferred to 1.5 mL tubes and DNA was eluted in 30 µL of pre-heated SE buffer (70°C) to maximize recovery.

For the DNeasy *mericon*® Food Kit, we applied the modified protocol of Zarzoso-Lacoste et al. (2013) for complex environmental matrices, which included an extended lysis (65°C, 4h, agitation), and an additional washing step using buffer AW2. DNA was eluted in 35 µL of AE buffer.

To minimize and control cross-sample contamination, each extraction series followed the contamination-control framework of Corse et al. (2017) and included:

- a maximum of 23 environmental DNA samples,
- one negative extraction control consisting of 500 µL of DNA-free water processed through the full extraction protocol, and
- one negative aerosol control consisting of a 1.5 mL vial containing 50 µL of DNA-free water left open but otherwise untouched during the extraction process.

Spectrophotometric quantification was performed using a NanoDrop™ One spectrophotometer (Thermo Fisher Scientific, MA, USA), and DNA concentrations were standardized to a maximum of 20 ng·µL⁻¹ with DNase/RNase-free water before PCR amplification.

**4. DNA quantification and normalization**

DNA yield and purity were further checked on a NanoDrop One spectrophotometer. Extracts were normalized to ≤ 20 ng·µL⁻¹ with DNase/RNase-free water and dispatched in 96-well plates. Plates were stored at 4 °C (for up to three days) or –20 °C (for longer storage) until COI enrichment.

**5. First PCR – COI enrichment**

COI amplicons were obtained using the IIICRrevN (3′-GGNTGAACNGTNTAYCCNCC-5′) and HBR2d (5′-TAWACTTCDGGRTGNCCRAARAAYCA-3′) primers, designed to capture a broad range of eukaryotic taxa (Thomasdotter et al., 2023). Forward and reverse primers were indexed with 11–13 nucleotide tags, each differing by at least three nucleotides to prevent misassignments (Corse et al., 2017). A 0–3 nucleotide heterogeneity spacer (none/N/NN/NNN) was added to COI primers to mitigate low-diversity sequencing issues (Fadrosh et al., 2014). Primers were complemented with Illumina adapter tails, and a unique tagged combination was used for each sample. PCRs were performed in 15 µL reactions with either the Qiagen Multiplex PCR Master Mix (Qiagen, Germany) or the Phusion® High-Fidelity PCR Master Mix (Thermo Fisher Scientific, MA, USA). Each reaction tubes contained 1.5 µL of each primer (2 µM), and 2 µL of DNA template.

For the Qiagen Multiplex PCR Master Mix, thermal cycling consisted of: 95 °C for 15 min; 5 cycles of 94 °C for 30 s, 45 °C for 40 s, 72 °C for 1 min; 30 cycles of 94 °C for 30 s, 48 °C for 40 s, 72 °C for 1 min; final extension at 72 °C for 10 min. While for the Phusion® High-Fidelity PCR Master Mix, thermal cycling consisted of 98 °C for 30 s, followed by 5 cycles of 98 °C for 10 s, 45 °C for 30 s, and 72 °C for 30 s; 30 cycles of 98 °C for 10 s, 55 °C for 30 s, and 72 °C for 30 s; final extension at 72 °C for 10 min.

All samples—including positive and negative controls—were amplified in triplicate, and PCR products were visualized on 2% agarose gels (0.5× TAE). Replicates were pooled by plate according to band intensity (1 µL for strong, 4 µL for medium, 10 µL for weak or absent bands). Each pool was purified using the Wizard® SV Gel and PCR Clean-Up System Kit (Promega, France).

**6. Second PCR – Indexing, library preparation, and sequencing**

A two-step tailed PCR approach was employed to generate paired-end, sequencing-ready libraries. The locus-specific COI primers contained Illumina-compatible sequence tails allowing the addition of Nextera® XT indexed adapters (Illumina, CA, USA) in a second amplification step). Each purified PCR pool was amplified in 60 µL reactions containing 30 µL of Phusion® High-Fidelity PCR Master Mix (Thermo Fisher Scientific, MA, USA), 2.1 µL of each PCR2 primer (20 µM), 1.8 µL of DMSO (3%), and 6 µL of DNA template from the purified pool. The thermal cycling program included an initial denaturation at 98 °C for 30 s, followed by 12 cycles of 98 °C for 10 s, 55 °C for 30 s, and 72 °C for 30 s, with a final extension at 72 °C for 10 min. PCR products were visualized on 2% agarose gels (0.5× TAE), excised, and purified using the Wizard® SV Gel and PCR Clean-Up System Kit (Promega, France). Purified libraries were quantified and quality-checked on an Agilent 2100 Bioanalyzer (DNA 1000 Kit; Agilent, CA, USA) before equimolar pooling. Sequencing was performed on an Illumina MiSeq using v2 chemistry (2 × 250 bp paired-end; Illumina, CA, USA) runs.

**7. Bioinformatic processing**

Reads were processed using VTAM (González et al., 2023) under identical parameter settings for all treatments. Metrics included total reads, number of validated ASVs, α-diversity (richness, Shannon index), and taxonomic composition at major ranks (Metazoa *vs.* non-Metazoa). We recall that only ASVs present in at least two of the three PCR replicate (per DNA extract) were kept (and pooled) to characterize each metabarcoding sample (see main manuscript).

**8. Results of optimization**

The NucleoSpin® Soil Kit consistently yielded a broader diversity of non-metazoan taxa (algae, protists, fungi) without reducing metazoan richness compared to the DNeasy *mericon*® Food Kit (Figure B).

PCRs with the Qiagen Multiplex PCR Master Mix generated higher read counts and more validated ASVs than those amplified with the Phusion enzyme, while maintaining similar taxonomic composition and reproducibility (Figure C). There was no phylogenetic effect, i.e. the increase in variant number was similar in distinct taxonomic groups (not shown).

These results led to the adoption of the NucleoSpin® Soil Kit and Qiagen Multiplex PCR Master Mix for all subsequent COI metabarcoding analyses of ARMS samples.

In addition, this test provides a cross-validation of our metabarcoding globally (across all four methods) owing to numerous metabarcoding replicates from the same community (up to 12) (Figure C). In a nMDS based on community similarity (same methods as in the main manuscript), samples clustered very well by biological samples, despite all biological samples come from Marseille, and some from the very same ARMS unit, cf. TableA). Thus, although we found differences among protocols, all replicates, including from distinct protocols, provide a rather similar picture of the community they come from.

**9. References**

Corse, E., Meglécz, E., Archambaud, G., Ardisson, M., Martin, J., Tougard, C., Chappaz, R., & Dubut, V. (2017). A from‐benchtop‐to‐desktop workflow for validating HTS data and for taxonomic identification in diet metabarcoding studies. *Molecular Ecology Resources*, 17(6), e146-e159. https://doi.org/10.1111/1755-0998.12703

Fadrosh, D. W., Ma, B., Gajer, P., Sengamalay, N., Ott, S., Brotman, R. M., & Ravel, J. (2014). An improved dual-indexing approach for multiplexed 16S rRNA gene sequencing on the Illumina MiSeq platform. *Microbiome*, 2(1), 6.

González, A., Dubut, V., Corse, E., Mekdad, R., Dechatre, T., Castet, U., Hebert, R., & Meglécz, E. (2023). VTAM: A robust pipeline for validating metabarcoding data using controls. *Computational and Structural Biotechnology Journal*, 21, 1151–1156. https://doi.org/10.1016/j.csbj.2023.01.034

Thomasdotter, A., Shum, P., Mugnai, F., Vingiani, M., Dubut, V., Marschal, F., Abbiati, M., Chenuil, A., & Costantini, F. (2023). Spineless and overlooked: DNA metabarcoding of autonomous reef monitoring structures reveals intra‐ and interspecific genetic diversity in Mediterranean invertebrates. *Molecular Ecology Resources*, 23(7), 1689–1705. https://doi.org/10.1111/1755-0998.13836

Zarzoso‐Lacoste, D., Corse, E., & Vidal, E. (2013). Improving PCR detection of prey in molecular diet studies: Importance of group‐specific primer set selection and extraction protocol performances. *Molecular Ecology Resources*, 13(1), 117-127.

**Table A: Sample metadata.**

| Sample | Bio Sample **^a^** | Nb_ASVs_ afterVTAMfilter | year | site | fieldReplicate  (=ARMS nb) | Fraction | Extr | Taq |
| --- | --- | --- | --- | --- | --- | --- | --- | --- |
| TEST1-MF201Phu | 1.2 | 44 | 2018 | MAR2 | 1 | 4B | MF2 | Phu |
| TEST1-MF201QM | 1.2 | 57 | 2018 | MAR2 | 1 | 4B | MF2 | QM |
| TEST1-MF202Phu | 1.2 | 14 | 2018 | MAR2 | 1 | 4B | MF2 | Phu |
| TEST1-MF202QM | 1.2 | 60 | 2018 | MAR2 | 1 | 4B | MF2 | QM |
| TEST1-MN01Phu | 1.2 | 20 | 2018 | MAR2 | 1 | 4B | MN | Phu |
| TEST1-MN01QM | 1.2 | 56 | 2018 | MAR2 | 1 | 4B | MN | QM |
| TEST1-MN02Phu | 1.2 | 25 | 2018 | MAR2 | 1 | 4B | MN | Phu |
| TEST1-MN02QM | 1.2 | 59 | 2018 | MAR2 | 1 | 4B | MN | QM |
| TEST1-MF203Phu | 3 | 36 | 2018 | MAR4 | 3 | MIX | MF2 | Phu |
| TEST1-MF203QM | 3 | 69 | 2018 | MAR4 | 3 | MIX | MF2 | QM |
| TEST1-MN03Phu | 3 | 17 | 2018 | MAR4 | 3 | MIX | MN | Phu |
| TEST1-MN03QM | 3 | 92 | 2018 | MAR4 | 3 | MIX | MN | QM |
| TEST1-MF104Phu | 4.5 | 43 | 2018 | MAR2 | 2 | 500 | MF1 | Phu |
| TEST1-MF104QM | 4.5 | 39 | 2018 | MAR2 | 2 | 500 | MF1 | QM |
| TEST1-MF105Phu | 4.5 | 57 | 2018 | MAR2 | 2 | 500 | MF1 | Phu |
| TEST1-MF105QM | 4.5 | 78 | 2018 | MAR2 | 2 | 500 | MF1 | QM |
| TEST1-MF204Phu | 4.5 | 61 | 2018 | MAR2 | 2 | 500 | MF2 | Phu |
| TEST1-MF204QM | 4.5 | 82 | 2018 | MAR2 | 2 | 500 | MF2 | QM |
| TEST1-MF205Phu | 4.5 | 64 | 2018 | MAR2 | 2 | 500 | MF2 | Phu |
| TEST1-MF205QM | 4.5 | 66 | 2018 | MAR2 | 2 | 500 | MF2 | QM |
| TEST1-MN04Phu | 4.5 | 54 | 2018 | MAR2 | 2 | 500 | MN | Phu |
| TEST1-MN04QM | 4.5 | 66 | 2018 | MAR2 | 2 | 500 | MN | QM |
| TEST1-MN05Phu | 4.5 | 51 | 2018 | MAR2 | 2 | 500 | MN | Phu |
| TEST1-MN05QM | 4.5 | 54 | 2018 | MAR2 | 2 | 500 | MN | QM |
| TEST1-MF106Phu | 6.7 | 24 | 2018 | MAR2 | 2 | 100 | MF1 | Phu |
| TEST1-MF106QM | 6.7 | 118 | 2018 | MAR2 | 2 | 100 | MF1 | QM |
| TEST1-MF107Phu | 6.7 | 38 | 2018 | MAR2 | 2 | 100 | MF1 | Phu |
| TEST1-MF107QM | 6.7 | 119 | 2018 | MAR2 | 2 | 100 | MF1 | QM |
| TEST1-MN06Phu | 6.7 | 39 | 2018 | MAR2 | 2 | 100 | MN | Phu |
| TEST1-MN06QM | 6.7 | 131 | 2018 | MAR2 | 2 | 100 | MN | QM |
| TEST1-MN07Phu | 6.7 | 0 | 2018 | MAR2 | 2 | 100 | MN | Phu |
| TEST1-MN07QM | 6.7 | 111 | 2018 | MAR2 | 2 | 100 | MN | QM |
| TEST1-MF208Phu | 8 | 1 | 2018 | MAR2 | 3 | MIX | MF2 | Phu |
| TEST1-MF208QM | 8 | 13 | 2018 | MAR2 | 3 | MIX | MF2 | QM |
| TEST1-MN08Phu | 8 | 2 | 2018 | MAR2 | 3 | MIX | MN | Phu |
| TEST1-MN08QM | 8 | 10 | 2018 | MAR2 | 3 | MIX | MN | QM |
| TEST1-MF209Phu | 9.10 | 46 | 2018 | MAR3 | 1 | MIX | MF2 | Phu |
| TEST1-MF209QM | 9.10 | 65 | 2018 | MAR3 | 1 | MIX | MF2 | QM |
| TEST1-MN09Phu | 9.10 | 5 | 2018 | MAR3 | 1 | MIX | MN | Phu |
| TEST1-MN09QM | 9.10 | 64 | 2018 | MAR3 | 1 | MIX | MN | QM |
| TEST1-MF210Phu | 9.10 | 27 | 2018 | MAR3 | 1 | MIX | MF2 | Phu |
| TEST1-MF210QM | 9.10 | 50 | 2018 | MAR3 | 1 | MIX | MF2 | QM |
| TEST1-MN10Phu | 9.10 | 32 | 2018 | MAR3 | 1 | MIX | MN | Phu |
| TEST1-MN10QM | 9.10 | 72 | 2018 | MAR3 | 1 | MIX | MN | QM |

**^a^** : The dots within Bio Sample names indicate that two separates DNA extractions were done for each extraction method, from the same biological sample. DNA extractions were named 1 to 10 for each method, at least one with the DNeasy *mericon*® Food Kit (MF1 and/or MF2) and one with the NucleoSpin® Soil Kit (MN)

**Figure A: Non-metric multidimensional scaling (nMDS) based on Bray-Curtis dissimilarities among samples.**

Symbols represent individual biological samples. The analysis was performed on ASV abundance data.

**Figure B: Taxonomic richness (number of ASVs) obtained with the two DNA extraction methods.**

Samples on the left (red background) were extracted with the DNeasy *mericon*® Food Kit (“kit MF”), and those on the right (green background) with the NucleoSpin® Soil Kit (“kit MN”). All results are based on metabarcoding amplified with the Qiagen Multiplex polymerase.

**Figure C: Rarefaction curves of ASV richness for the four extraction–polymerase combinations.**

The horizontal axis represents the number of samples and the vertical axis (log scale) represents the number of ASVs. Light colours correspond to the DNeasy *mericon*® Food Kit and dark colours to the Nucleospin® Soil Kit. Blue curves indicate amplifications performed with the Qiagen Multiplex PCR Master Mix, and red curves those using the Phusion® High-Fidelity PCR Master Mix.
